# Human-centered evaluation of explainable AI applications: a systematic review

**DOI:** 10.3389/frai.2024.1456486

**Published:** 2024-10-17

**Authors:** Jenia Kim, Henry Maathuis, Danielle Sent

**Affiliations:** ^1^HU University of Applied Sciences Utrecht, Research Group Artificial Intelligence, Utrecht, Netherlands; ^2^Jheronimus Academy of Data Science, Tilburg University, Eindhoven University of Technology, 's-Hertogenbosch, Netherlands

**Keywords:** explainable AI, XAI, human-centered evaluation, meaningful explanations, XAI evaluation, systematic review, human-AI interaction, human-AI performance

## Abstract

Explainable Artificial Intelligence (XAI) aims to provide insights into the inner workings and the outputs of AI systems. Recently, there's been growing recognition that explainability is inherently human-centric, tied to how people perceive explanations. Despite this, there is no consensus in the research community on whether user evaluation is crucial in XAI, and if so, what exactly needs to be evaluated and how. This systematic literature review addresses this gap by providing a detailed overview of the current state of affairs in human-centered XAI evaluation. We reviewed 73 papers across various domains where XAI was evaluated with users. These studies assessed what makes an explanation “good” from a user's perspective, i.e., what makes an explanation *meaningful* to a user of an AI system. We identified 30 components of meaningful explanations that were evaluated in the reviewed papers and categorized them into a taxonomy of human-centered XAI evaluation, based on: (a) the contextualized quality of the explanation, (b) the contribution of the explanation to human-AI interaction, and (c) the contribution of the explanation to human-AI performance. Our analysis also revealed a lack of standardization in the methodologies applied in XAI user studies, with only 19 of the 73 papers applying an evaluation framework used by at least one other study in the sample. These inconsistencies hinder cross-study comparisons and broader insights. Our findings contribute to understanding what makes explanations meaningful to users and how to measure this, guiding the XAI community toward a more unified approach in human-centered explainability.

## 1 Introduction

The past decade has shown an exponential growth of Artificial Intelligence (AI) across all sectors of society, including healthcare, finance, and education. In many sectors, high-risk decision-making tasks are readily available, such as medical diagnosis or credit scoring, in which the outcomes could have severe consequences for individuals (Ngai et al., 2011; Bright et al., 2012; Antoniadi et al., 2021; Umbrello and Yampolskiy, 2022; Souza and Leung, 2021). Due to these consequences, integration of an AI-based system into a decision-making process requires understanding how it reaches a certain prediction or recommendation.

Broadly speaking, there are two types of AI models: black-box models and white-box models. White-box models are characterized by their transparency and interpretability. Users can inspect the white-box model's internal mechanisms and comprehend the rationale behind its predictions. Black-box models, on the other hand, refer to algorithms or systems in which the internal workings and processes are opaque and not (easily) interpretable.

Due to their complexity and non-linearity, black-box models usually offer superior predictive performance over white box-box models, making them an attractive choice in the current AI landscape. However, they pose challenges in terms of interpretability, accountability, and trust (Rai, 2020; Diakopoulos, 2014). Responsible integration of black-box models into decision-making processes necessitates some level of transparency regarding their reasoning and workings, for a few reasons. First, it is needed for regulatory compliance; for example the European Union General Data Protection Regulation (GDPR) requires that “*meaningful information about the logic involved”* is provided to people who are affected by automated decision-making systems (Article 13). Second, black-box models might exhibit biases or make decisions based on irrelevant or spurious correlations in the data, leading to unintended consequences or ethical concerns. Third, successful adoption of a model by the intended users requires that they comprehend and trust the model's decisions. In response to these needs, Explainable Artificial Intelligence (XAI) has emerged to provide insights into the inner workings of (opaque) AI systems.

The foundational work in XAI focused predominantly on the technical aspects of generating explanations of black-box models. Evaluation of XAI focused mainly on the objective quality of the generated explanations, for example their correctness (whether they faithfully describe the workings of the black-box model) and completeness (how much of the black-box behavior they describe). This aspect of XAI evaluation can be viewed as **computer-centered** (Lopes et al., 2022), and can be associated with the perspective of the system's developers, who need to “*look at a given explanation and make an a priori (or decontextualized) judgment as to whether or not it is good”* (Hoffman et al., 2023).^1^

However, in recent years, a growing recognition has emerged that explainability is an inherently human-centric property (Miller, 2019; Liao et al., 2020), which “*lies in the perception and reception of the person receiving the explanation”* (Liao and Varshney, 2021). That an explanation is a priori “good” (correct, complete, etc.) is not sufficient to make it effective, beneficial, or meaningful for the person interacting with the AI system; there are additional, **human-centered** components of explanation quality, which are essential to achieve goals like understanding, trust or good decision making. We refer to these human-centered aspects as “explanation meaningfulness.”

The shift toward a human-centered approach transformed XAI from a mostly technology driven field into a multidisciplinary research effort (Mohseni et al., 2021; Lopes et al., 2022). XAI research from a machine learning perspective focuses on the technical challenges of generating explanations of black-box models (or alternatively, designing high-performing inherently interpretable models) (e.g., Loyola-Gonzalez, 2019). XAI research in human-computer interaction (HCI) focuses on identifying and addressing the needs of the users who interact with the system and the explanations (e.g., Haque et al., 2023; Liao and Varshney, 2021; Ferreira and Monteiro, 2020). XAI from the cognitive science perspective examines how personality traits and cognitive biases affect the processing of explanations and their effectiveness (e.g., Bertrand et al., 2022). XAI research from the social science perspective looks into how people explain to each other and what social expectations might be involved in the processing of explanations (e.g., Borrego-D́ıaz and Galán-Páez, 2022).

This multidisciplinarity contributes to a rich and nuanced exploration of XAI, which does justice to the complexity of the topic. However, it also brings about considerable challenges. One of the challenges is the lack of consensus within the research community regarding the evaluation of XAI (Lopes et al., 2022). First, despite the wide recognition that explainability serves a user need, empirical evaluation with users is not yet a standard practice (Anjomshoae et al., 2019; Nauta et al., 2023). Second, there is no consensus about which properties need to be evaluated to make sure that an explanation is meaningful to users, i.e. what are the specific components of human-centered explanation quality. Third, there are no standardized evaluation frameworks and procedures, which makes it difficult to interpret and compare results from different studies and build on previous body of knowledge. In other words, consensus is lacking regarding whether human-centered evaluation is a crucial component of XAI evaluation; and if so, what needs to be evaluated, and how it needs to be evaluated. Several recent studies addressed this gap by creating taxonomies of XAI evaluation based on systematic literature reviews (Mohseni et al., 2021; Vilone and Longo, 2021; Lopes et al., 2022; Jung et al., 2023; Nauta et al., 2023); our work builds upon and further extends these efforts.

We review 73 papers from different domains, which evaluate various aspects of what makes an explanation *meaningful* to a user interacting with an AI system. We provide a comprehensive overview of the evaluation methodology applied in these user studies; this allows us to identify what the XAI research community considers as the components of a meaningful explanation. In other words, this literature study addresses the research question: “*How is the meaningfulness of XAI explanations evaluated in user studies?”*; the sub-questions are: (a) what aspects of human-centered explanation quality are evaluated in XAI user studies (evaluation measures), and (b) how are these aspects evaluated (evaluation procedures). Based on this analysis, we propose a new taxonomy of human-centered XAI evaluation, which categorizes the identified evaluation measures along three dimensions: (a) the contextualized quality of the explanation, (b) the contribution of the explanation to human-AI interaction, and (c) the contribution of the explanation to human-AI performance.

The contribution of this review is threefold. First, to the best of our knowledge, we provide the most detailed overview of the human-centered evaluation measures that are currently in-use in the XAI research community. This elaborate analysis emphasizes the fact that there are many different components that make an explanation meaningful to a user interacting with an AI system. It also highlights the lack of consensus in the research community regarding both the components and the terminology used to refer to them, which results in a multitude of partially overlapping constructs. Second, our proposed taxonomy offers a novel way of organizing human-centered evaluation measures, which highlights that meaningful explainability hinges not only on the quality of the explanation itself, but also on the role it plays in human-AI interaction and human-AI performance. This categorization scheme makes the main concepts of human-centered XAI more accessible for future research, which can help both with identifying gaps and with standardization of terminology. Third, this paper provides an overview of the existing standard frameworks (questionnaires, indices, scales) that are used to evaluate explanations in user studies. We observe that the majority of the studies create their own methodology, tailored to their specific use case; moreover, even in the few cases where the same framework is used, there is variation across the studies in how the evaluation procedure is applied. This lack of standard evaluation methodology makes it difficult to compare between studies and potentially discover insights and patterns beyond specific use cases. By making these inconsistencies explicit, this review can aid the XAI research community in making the necessary next steps toward a more unified approach, which can lead to a deeper level in the exploration of human-centered explainability.

The structure of this paper is as follows. In Section 2, we provide an overview of existing taxonomies of XAI evaluation to contextualize our research. Section 3 details the methodology employed in this literature study. In Section 4, we present the main findings: Section 4.1 reports selected statistics on the included papers; Section 4.2 introduces the 30 identified evaluation measures and the proposed taxonomy, and examines how our taxonomy relates to existing XAI evaluation frameworks; Section 4.3 discusses the evaluation procedures applied in the reviewed papers. Finally, Section 5 offers a comprehensive discussion of the findings.

## 2 Related work

One of the challenges that the field of XAI currently faces is the lack of consensus regarding the properties that make explanations “good” (in an a priori, decontextualized sense) and “meaningful” (to users, in the context of use), as well as lack of standardization in the evaluation methods that are applied to measure these properties. This has been observed in a few recent reviews, including Lopes et al. (2022) and Nauta et al. (2023). The first step in addressing this challenge is to inventorize the evaluation properties and methods that are already in use by the research community, and organize them in a conceptual framework, i.e., a taxonomy. A taxonomy provides a structure for organizing existing and new concepts, thus making them more accessible and facilitating the creation of a common terminology within the research domain.

This approach is applied in this work as well; we inventorize the existing evaluation measures for human-centered XAI evaluation and organize them into a taxonomy. Our work extends existing similar efforts, complementing them in some ways and diverging from them in others. In order to contextualize our contribution, this section discusses some prominent existing taxonomies for XAI evaluation that have been proposed in the literature, specifically by Doshi-Velez and Kim (2018), Hoffman et al. (2018, 2023), Zhou et al. (2021), Vilone and Longo (2021), Mohseni et al. (2021), Lopes et al. (2022), and Nauta et al. (2023).

**Figure 1:**
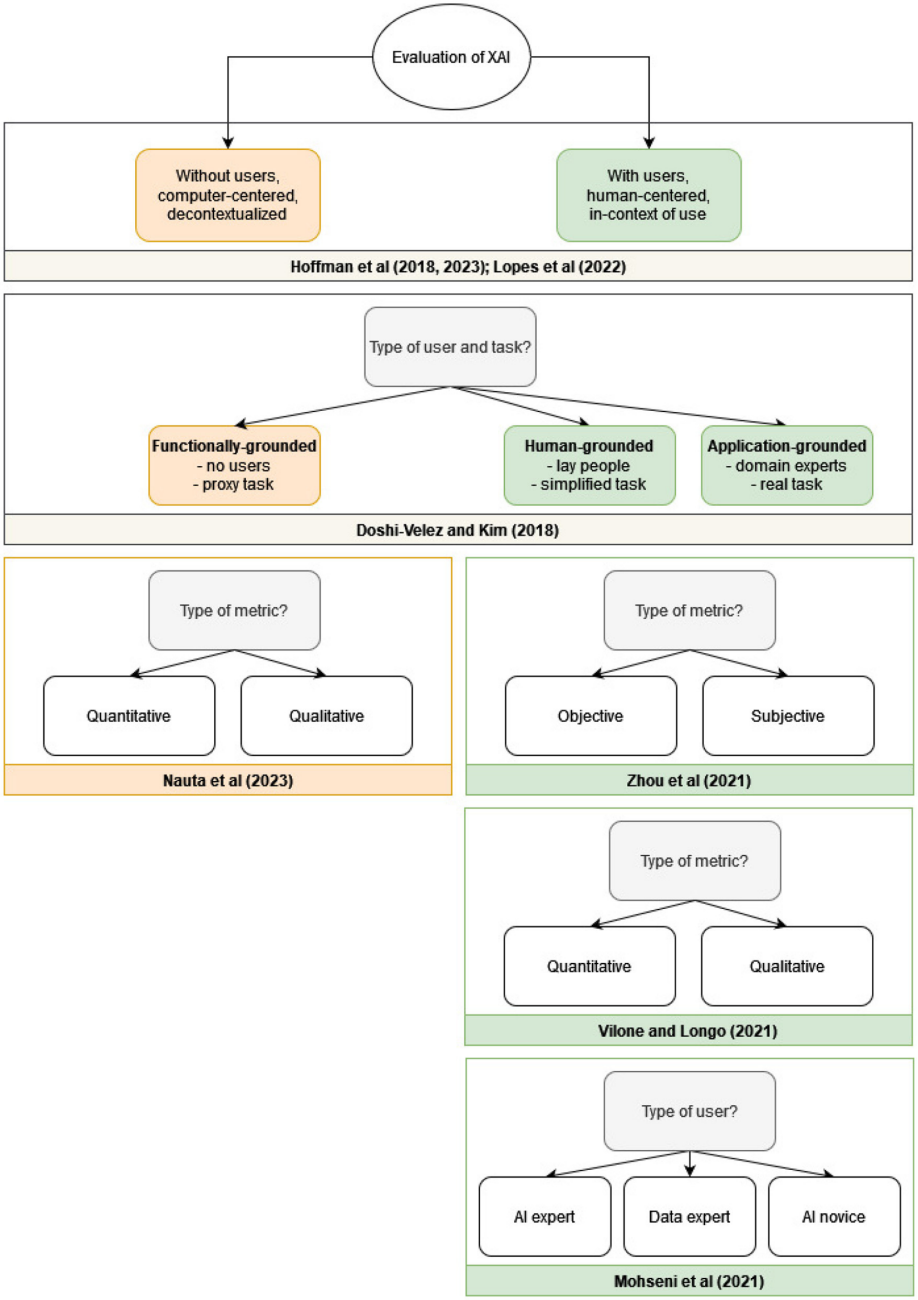
The high-level categories in existing taxonomies for XAI evaluation. Doshi-Velez and Kim (2018), Hoffman et al. (2018, 2023), and Lopes et al. (2022) discuss both evaluation with users (green) and without users (orange); Nauta et al. (2023) focus on evaluation without users (orange); Zhou et al. (2021), Vilone and Longo (2021), and Mohseni et al. (2021) focus on evaluation with users (green).

In all the discussed XAI evaluation taxonomies, the high-level distinction that is used (explicitly or implicitly) is between *human-centered evaluation* with users and *computer-centered evaluation*, which is conducted without human participants. This is shown in Figure 1, where the main (high-level) categories of each taxonomy are presented. Beyond this high-level categorization, some taxonomies focus on various additional aspects of the evaluation. Doshi-Velez and Kim (2018) propose a three-fold categorization scheme which is based on the type of task and the type of user involved. In their taxonomy, *functionally-grounded evaluation* does not involve experiments with human participants, *human-grounded evaluation* involves a simplified task with lay people as participants, and *application-grounded evaluation* involves experiments with a real task/application and the intended users of the application as participants.

Mohseni et al. (2021) proposes a different categorization of users: AI novices (users of AI systems who have little expertise in machine learning), data experts (domain experts who use machine learning for analysis, decision making, or research), and AI experts (machine learning scientists and engineers who design AI systems). Each of these groups is associated with different goals that need to be taken into account when designing an XAI system.

Focusing on human-centered evaluation, Zhou et al. (2021) extend the categorization further by distinguishing between two types of metrics: *subjective metrics*, which focus on the perception of the users (e.g. trust, satisfaction, preference, confidence, etc.), and *objective metrics*, in which task-related, physiological or behavioral indicators of the users are measured (e.g. time spent on task, accuracy of predicting the model's output, galvanic skin response, gaze fixation, percentage of response-switching to match the model's recommendation, etc.). Vilone and Longo (2021), on the other hand, focus on the distinction between *qualitative metrics* (such as open-ended questions) and *quantitative metrics* (such as ratings).

Nauta et al. (2023) also distinguishes between quantitative and qualitative evaluation. Their main focus is on functionally-grounded evaluation, without users. They call for integration of objective and quantitative evaluation metrics “*as optimization criteria during model training in order to optimize for accuracy and interpretability simultaneously”* (Nauta et al., 2023).^2^

In addition to the high-level categorization shown in Figure 1, each of the taxonomies further goes into categorization of specific evaluated properties (e.g. *trust, satisfaction*); this is discussed in detail in Section 4.2.3.

## 3 Methodology

This literature study addresses the research question: “*How is the meaningfulness of XAI explanations evaluated in user studies?”*. To achieve this, we systematically identified studies that evaluate XAI with users, and extracted detailed information about the evaluation methodology applied in them. The method applied for identification, selection and analysis of relevant papers is described in the next sections.

### 3.1 Literature search

The goal of the search was to systematically identify studies that evaluate XAI with users, i.e., human-centered XAI evaluation. Finding the right keywords to capture this subset of XAI literature was not straightforward and required a few iterations of trial and optimization. Some keywords that we tried, e.g. users, proved to be too restrictive and were therefore removed from the search string. Other keywords, e.g. evaluation, proved to be too broad. The final search string that we arrived at after several iterations contained two elements: (1) a mention in the abstract of XAI, explainable AI, explainable artificial intelligence, or explainable machine learning, and (2) a mention in the abstract of words related to human perception of explanations: meaningful, trust*, understandable or interpretable. The human-centered keywords were found through an iterative process of identifying relevant papers and searching which words in their abstracts indicated specifically toward evaluation with users.

**Figure 2:**
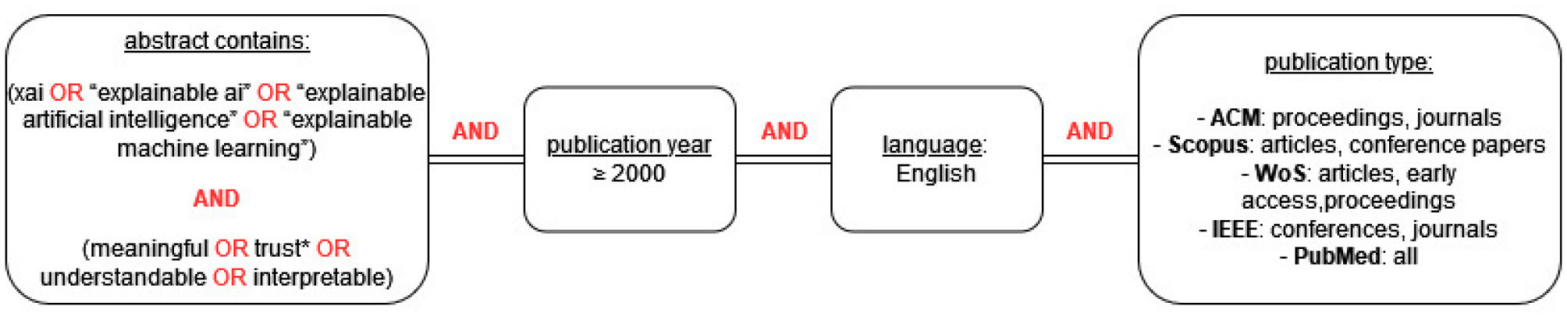
Search query for the systematic literature review.

Our goal was to provide a broad overview, not restricted to specific disciplines; even though the content and context of explanations might vary significantly per domain, evaluation measures and methods are not domain-specific (e.g., whether an explanation is experienced by users as understandable or trustworthy is relevant across disciplines). Therefore, we searched in: ACM Digital Library, Scopus, Web of Science, IEEE Xplore and PubMed. In each database, the search was restricted to peer-reviewed publication types; in addition, the search was limited to studies in English, published after the year 2000. The exact query is shown in Figure 2.

The search in all databases was performed in November 2023 and resulted in a total of 3,103 papers (ACM: 297; Scopus: 1,501; WoS: 614; IEEE: 381; PubMed: 310). After removing the duplicates, 1,655 papers remained.

### 3.2 Selection process

**Figure 3:**
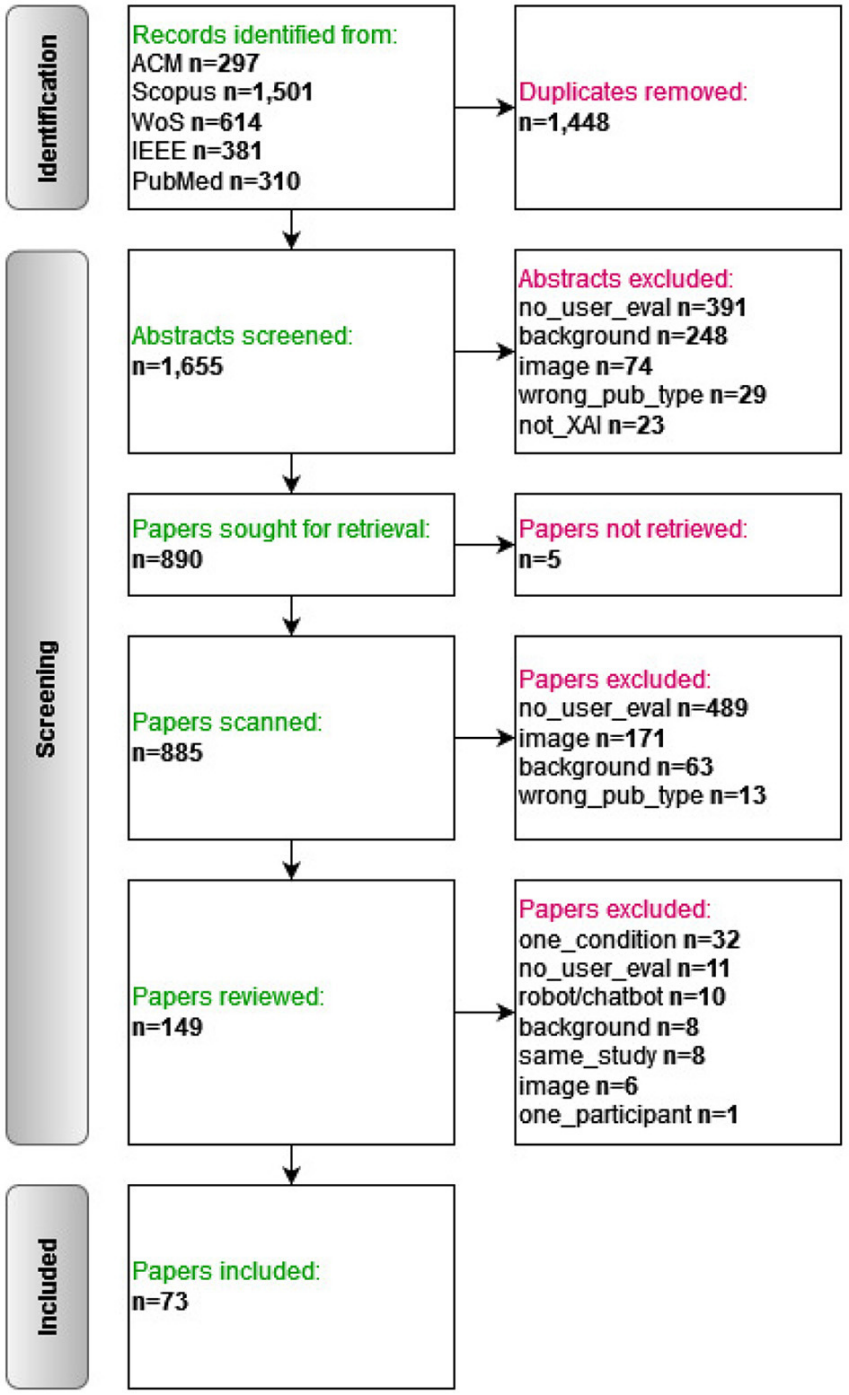
PRISMA flow diagram.

The selection of the papers was performed in three rounds: (1) abstract screening, (2) full text screening, and (3) full text review. The PRISMA information flow diagram of the whole process is presented in Figure 3.

Papers that fulfilled one of the following exclusion criteria were excluded (the labels in parentheses are the ones shown in Figure 3):

**Exclusion criteria:**

- Theoretical paper/literature study/review/etc. (“background”).
- No evaluation with users (“no user eval”).
- Image data; explanations in the form of heatmaps etc. (“image”).
- Agent explaining its actions/failures (“robot/chatbot”).
- Evaluation with one participant (“one participant”).
- Evaluation of explanations in a single condition (no baseline). (“one condition”).
- Not about Explainable AI (“not XAI”).
- Wrong publication type, e.g. letter, editorial (“wrong publication type”).
- Another paper describing the same user study is already included (“same study”).

In the first stage, we read all the abstracts (*n* = 1,655) and excluded those which fulfilled one of the exclusion criteria. This was performed by two researchers in a double-blinded manner (all three authors were involved in several duo compositions); discrepancies were periodically discussed and resolved with a third reviewer that was not part of the original duo reviewing the paper. In the second stage, the full texts of the remaining papers were retrieved (*n* = 885); two double-blinded researchers scanned the papers, focusing specifically on the methodology section, and checking whether evaluation with users was part of the study. In the third stage, the remaining papers (*n* = 149) were reviewed in detail for data extraction; each paper was reviewed by one researcher only, but excluded papers were discussed with a second researcher. In case the reviewers did not arrive at the same decision, a third reviewer made the final decision.

The selection process resulted in 73 papers which fulfill the following inclusion criteria:

**Inclusion criteria:**

- Studies that involve a decision support AI system, which provides recommendations/predictions and explanations.
- The research includes evaluation of the explanations with a user study.
- The user study includes more than one participant.
- The user study compares between at least two conditions (explanation vs. no explanation, alternative explanation types, or alternative explanation formats).

The choices regarding the inclusion and exclusion criteria were guided by various considerations. First, we only included studies that compare between at least two conditions; this was due to the fact that in the broader context of the project of which this literature study is a part, we were interested in finding patterns regarding user preferences toward specific types and/or formats of explanations. Second, we only included studies with systems based on tabular and textual input data; we excluded studies that focus on image data or on autonomous agents (robot/chatbot) that explain their actions/failures. This was primarily a scoping consideration; systems based on image data and autonomous agents have distinct explanation formats and explainability objectives, therefore it seems reasonable to treat them separately in future work. Third, we included studies where the AI system and/or the XAI system are a mock-up rather than a real algorithm (the so-called *Wizard of Oz experiment*), since such studies can still provide insights about user preferences, even if these explanations are not automatically generated.

### 3.3 Data extraction and analysis

**Figure 4:**
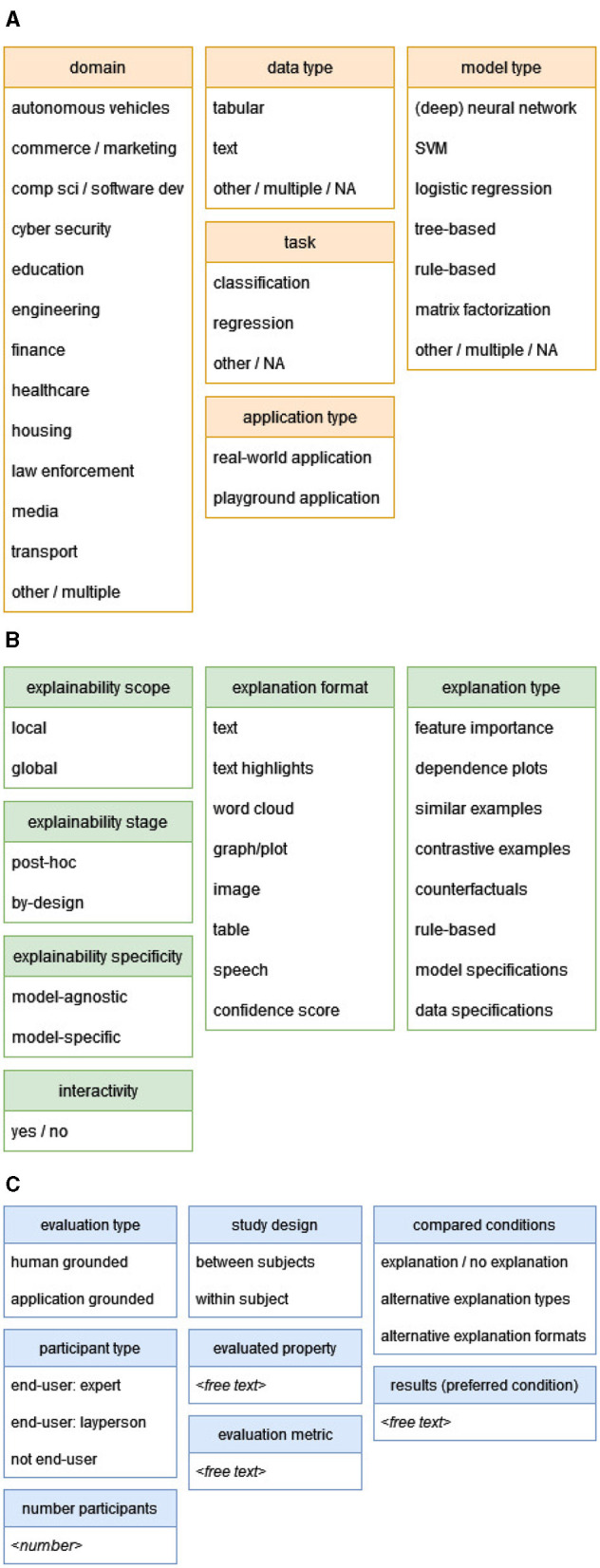
The labeling scheme used for data extraction. **(A)** Labeling scheme of AI systems. **(B)** Labeling scheme of XAI methods. **(C)** Labeling scheme of user studies.

After full text screening, the 73 included papers were labeled according to pre-defined categories, as shown in Figure 4. From each paper, we extracted detailed information about the characteristics of the AI system, the XAI method, and the user study. These categories and labels are mainly based on taxonomies in other XAI reviews (e.g., Nauta et al., 2023; Chromik and Schuessler, 2020), and adapted to what was actually observed in our set of included papers.

#### 3.3.1 Labeling scheme of AI systems

For the AI application/system (Figure 4A), we extracted the domain (e.g., healthcare, finance), the type of algorithm used (e.g., SVM, neural network), the type of input data (e.g., tabular, text), the type of task (e.g., classification, regression), and whether the application is real-world or playground. The latter distinction refers to whether the AI system addresses a real-world problem, or rather the task itself is of little importance and the focus of the research is on user behavior, for example preference toward one type of explanation rather than another.^3^

#### 3.3.2 Labeling scheme of XAI methods

For the XAI method (Figure 4B), we extracted the scope of the provided explanations, distinguishing between local explanations, which clarify individual predictions, and global explanations, which offer insights into the model's overall behavior. We also considered the explainability stage, differentiating between methods that are interpretable by-design (inherently interpretable models) and those that are *post-hoc* (applied after model training to interpret complex models). Additionally, we evaluated the specificity of the XAI method, identifying whether it is model-agnostic (applicable to any model) or model-specific (tailored to particular model types). For more details about the dichotomies described above, the reader is referred to Linardatos et al. (2020).

The type of the explanations was also labeled; the types we observed include feature importance (highlighting key features influencing predictions), dependence plots (showing relations or interactions between features), similar examples (comparing similar instances with the same prediction), contrastive examples (similar examples with a different prediction), counterfactuals (showing minimal changes needed to alter outcomes), rule-based explanations (providing human-readable rules), model specifications (explaining model workings or providing performance metrics), and data specifications (information about the training data). Finally, we noted whether the user can interact with the explanation, for example by requesting more or less details.

#### 3.3.3 Labeling scheme of user studies

For the user studies (Figure 4C), we extracted:

- Information about the study design, including the setup (between-subjects or within-subjects), and the compared conditions (explanation vs. no explanation, alternative explanation types, alternative explanation formats);
- Information about the participants, including their number and type (see below);
- Information about the evaluation methodology: type of evaluation (human-grounded or application-grounded; based on Doshi-Velez and Kim, 2018, see also Section 2), the evaluated property (e.g., the user's trust in the AI system), the metric used (e.g., ratings), and the results (which condition was preferred; e.g., users rated feature importance explanations as more trustworthy than similar examples).

For participants' type, we distinguish between *end-users* and *not end-users*. With the term *end-user* we refer to a person who uses the AI system, i.e. interacts directly with its outputs as part of a decision making process and needs to validate the AI reasoning. This does not include other stakeholders of XAI (mentioned in e.g. Meske et al., 2022), like the people affected by AI-based decisions, the people responsible for compliance of AI systems in the organization, AI regulators, and AI developers.^4^ When the participants of the user study are the intended users of the AI system, they are considered *end-users*; those end-users can be *expert end-users* (for example, doctors testing a clinical decision support system) or *lay end-users* (for example, social media users testing a fake news detection system). On the other hand, when the participants of the user study are not the intended users of the AI system, they are categorized as *not end-users* (for example, laypersons testing a clinical decision support system).

## 4 Results

In this section, we present our main findings regarding the research question “*How is the meaningfulness of XAI explanations evaluated in user studies?”*. Two topics are addressed:

- **What components of meaningfulness are evaluated in the user studies?** In Section 4.2, we present the 30 components of a meaningful explanation that were identified in the papers. We organize the components into a taxonomy of human-centered XAI evaluation, along three dimensions: *the in-context quality of the explanation, the contribution of the explanation to human-AI interaction*, and *the contribution of the explanation to human-AI performance*. We show which dimensions and components are most commonly evaluated in the set of papers we analyzed. Finally, we describe how the taxonomy relates to existing frameworks for XAI evaluation.
- **How are the components of meaningfulness evaluated?** In Section 4.3, we discuss the evaluation methodologies applied in the set of papers, and show that currently there is no standardized approach to XAI evaluation with end-users.

In order to contextualize these findings, we first present statistics about the papers included in the set (Section 4.1): the publication year, the application domain, the types of explanations discussed, and the type and number of participants in the described user studies.

### 4.1 Descriptive statistics

#### 4.1.1 Publication year

Table 1 shows the distribution by publication year of all the papers found by our query (after deduplication, *N* = 1,655), and the included papers (*N* = 73). As evident from the table, there are few papers matching the query that were published before 2018 (three out of 1,655). None of these papers ended up in the included set. From 2018 onwards, we observe a consistent increase in the number of papers matching the query; however, the majority of the included papers (68 out of 73; 93%) were published in 2021 onwards. This suggests that even though the field of XAI has been on a constant rise since 2018, the evaluation of explanations through user studies (human-centered evaluation) is a relatively recent development in the field.

**Table 1:** Distribution by publication year of the initially identified papers and the included papers (query date: 16-Nov-2023).

**Year**	***N* identified papers**	***N* included papers**
2005–2017	3	0
2018	35	0
2019	58	1
2020	138	4
2021	291	20
2022	493	20
2023 (Jan–Nov)	637	28
Total	1,655	73

#### 4.1.2 Application domain

**Figure 5:**
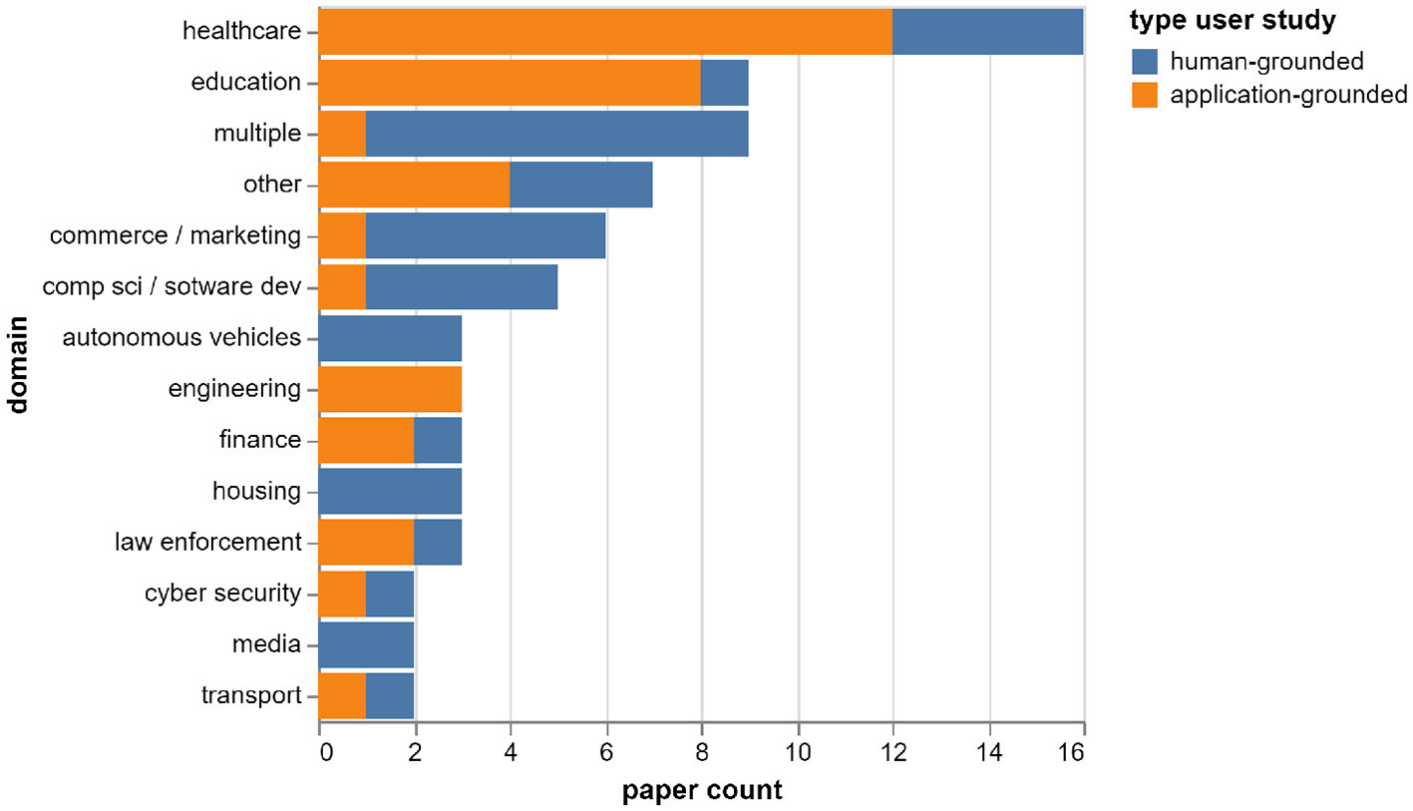
Distribution of papers by application domain and type user study.

Figure 5 shows the distribution of the included papers by application domain, and the proportion of human-grounded vs. application-grounded evaluations in each domain. The most common domains in the sample are healthcare (16 papers) and education (nine papers). In these domains, most of the user studies are performed in an application-grounded setting, i.e. with a real task and the intended end-users of the application (healthcare: 12 out of 16 papers, education: eight out of nine papers). However, in the overall set, only half of the studies (36 out of 73) are performed in an application-grounded setting; the other half is evaluated with lay people, and involves either a simplified task or a playground application (for which there are no real intended users).

#### 4.1.3 Explanation scope and type

The majority of the included studies (51 out of 73; 70%) focus on local explanations, i.e. those that explain an individual instance of the model's output (prediction/recommendation), rather than the entire model/system. There are only seven studies (10%) that focus on global explanations, and 15 studies (21%) that consider both local and global explanations.

In terms of specific explanation types, *feature importance* is the most commonly used explanation in the set (discussed in 44 out of the 73 papers; 60%), followed by rule-based explanations, which are discussed in 20 out of the 73 papers (27%) (if we include mock-up rule-based explanations in the count, it is 30 out of 73 papers; 41%). Additional details about the distribution of explanation types in the analyzed set of papers (including a full list of which explanation types are discussed in which paper) can be found in the Supplementary material.

#### 4.1.4 Participants

Table 2 shows information about the type and number of participants in the 77 user studies described in the 73 papers in our set. 26 out of the 77 user studies (34%) were conducted with expert end-users, such as healthcare professionals, teachers, law enforcement officers, etc. 18 out of the 77 user studies (23%) were conducted with lay end-users (non-professionals who are the intended users of the application), such as online chess players, social media users, patients, etc. 33 out of the 77 user studies (43%) were conducted with lay people who are not the intended end-users of the application; usually, these are participants recruited through the various online crowd-sourcing platforms.

**Table 2:** Number of participants (mean, min, max) and number of studies per participant type.

**Type participant**	***n* user studies**	***n*_*mean*_ participants**	***n*_*min*_ participants**	***n*_*max*_ participants**
End-user: Expert	26	24	2	124
End-user: Layperson	18	141	11	500
Not end-user	33	165	5	1343

In terms of number of participants, user studies with experts usually have fewer participants; the mean in our sample is 24 participants per study, the minimum is two participants, and the maximum is 124 participants. Studies with lay people tend to be bigger; the mean for lay end-users is 141 per study (with a maximum of 500), and the mean for non-end-users is 165 per study (with a maximum of 1,343).

### 4.2 The components of meaningfulness evaluated in user studies

In this section we present the findings regarding the components of a *meaningful explanation*, based on analysis of the evaluation methodology in the set of included papers. First, we show the taxonomy of human-centered evaluation of XAI, which we constructed based on the 30 components of meaningfulness found in the set of papers (Section 4.2.1). Next, the distribution of the components in the papers is presented (Section 4.2.2). Finally, we relate and map our proposed taxonomy to existing frameworks of XAI evaluation (Section 4.2.3).

#### 4.2.1 Taxonomy of human-centered evaluation of XAI

**Figure 6:**
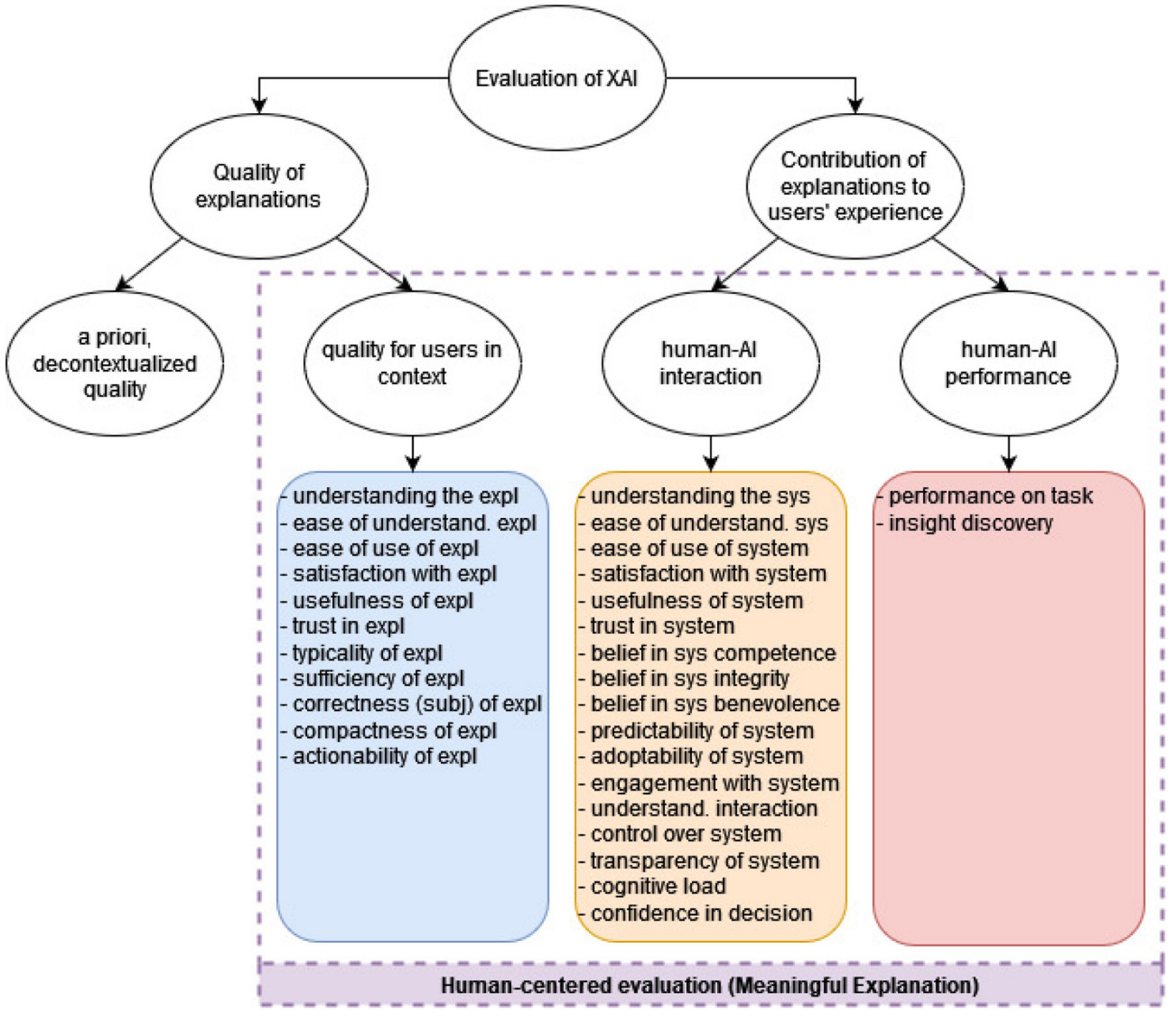
The proposed taxonomy of human-centered evaluation of XAI. The blue, orange and red boxes contain the 30 evaluation measures identified in the reviewed papers. They are grouped based on the aspect of human-centered explanation quality that they evaluate: **(A)** in-context quality of the explanation, **(B)** contribution of the explanation to human-AI interaction, and **(C)** contribution of the explanation to human-AI performance. An additional aspect, the a priori explanation quality, is not covered by our review since it is not evaluated with users (expl = explanation; sys = system).

From the set of 73 included papers, we systematically collected 30 properties that were evaluated in the user studies. These properties were then grouped into a taxonomy for human-centered evaluation of XAI, shown in Figure 6.

The highest-level category in our taxonomy addresses the question: *What is a good explanation of an AI system?*. This involves two aspects: the **quality of the explanation** itself (whether it is correct, complete, understandable, actionable, etc.), and the **contribution of the explanation to the users' experience of using the AI system** (e.g., whether the explanation improves the user's understanding of the AI system, or contributes to a better performance on the task at hand).

The quality of explanations is further categorized into two types. The first one is the **a priori, decontextualized explanation quality**, which is evaluated by the system developers prior to the introduction of the system into the intended context of use; this includes, for example, evaluation of the explanation's objective correctness (i.e. whether the explanation faithfully describes the workings of the predictive model). The second one is the **in-context quality of the explanation** that is experienced by the user, like its understandability and usefulness. These two aspects are not necessarily aligned; for example, an explanation can be objectively incorrect (does not represent the model faithfully), but still be experienced as understandable, trustworthy and sufficient by a user.

The contribution of explanations to users' experience is also categorized into two dimensions: the contribution of the explanation to **human-AI performance** and to **human-AI interaction**. Those two aspects are closely related and both have to do with human-AI collaboration; the difference is that *performance* is focused on the results and outputs of the collaboration, while *interaction* is concerned with the experience of working with the AI system and on the system's perceived quality from the perspective of the user. For example, a user can experience that explanations improve the interaction with the AI system, because they make the system more understandable and trustworthy; at the same time, the user's actual performance on the task might not be influenced by the presence of explanations.

Out of the four dimensions discussed so far, one (a priori, decontextualized explanation quality) is *computer-centered*, and the other three are *human-centered*. In our systematic literature review, we focused on human-centered evaluation with user studies; therefore, the 30 properties that we identified are categorized along these three dimensions. As shown in Figure 6, 11 properties were identified that evaluate the in-context quality of explanations (blue box); for example, explanation's understandability, usefulness, sufficiency, trustworthiness, actionability etc. Furthermore, 17 properties were identified that evaluate the contribution of explanations to human-AI interaction (orange box); for example, the contribution of explanations to the transparency of the AI system, to the trustworthiness of the system, to the perceived control over the system, etc. Lastly, two properties were identified that evaluate the contribution of explanations to human-AI performance (red box): the contribution of explanations to the performance on the task at hand, and to insight discovery.

The 30 properties identified in the studies can be measured in various ways: quantitatively or qualitatively, objectively or subjectively. For example, the contribution of explanations to the *understandability of the AI system* can be measured subjectively by asking the participants how well do they understand the system; responses can be collected either through ratings (quantitative) or free text (qualitative). Arguably, it can be also measured objectively and quantitatively by, for example, asking the participants to predict the model's output and calculating their accuracy score. These distinctions, which are central to the existing taxonomies as described in Section 2, are important from a methodological point of view; however, we do not view them as the main categories of XAI evaluation, and therefore did not include them in our taxonomy as shown in Figure 6. The full taxonomy of the metrics identified in the set of 73 papers (including their categorization into objective vs. subjective and quantitative vs. qualitative metrics), as well as the detailed distribution of the metrics in the set of papers, can be found in the Supplementary material.

In conclusion, the human-centered evaluation of XAI in the studies we analyzed is performed along three dimensions:

- The in-context quality of the explanation (11 components). Is the explanation *satisfying, understandable, useful, actionable, sufficient, compact, trustworthy, correct, typical, easy to understand*, and *easy to use*?
- The contribution of the explanation to human-AI interaction (17 components). Does the explanation help the user to better *understand the AI system*? Does it improve the user's perception of the AI system as *trustworthy, useful, satisfying, competent, honest, benevolent, controllable, predictable, transparent, easy to understand, easy to use*, and *engaging*? Does the explanation help the user to better *understand the interaction with the AI system*? Does it make the interaction less *cognitively demanding*? Does it increase the user's *confidence in the decision*? Does it increase the *readiness to adopt the AI system* and use it?
- The contribution of the explanation to human-AI performance (two components). Does the explanation improve the user's *performance on the task*? Does it help the user to *discover new insights*?

The taxonomy is described in more detail in Section 4.2.3, where it is placed in the context of existing taxonomies for XAI evaluation.

#### 4.2.2 Distribution of the components in the papers

Table 3 shows which of the three dimensions of human-centered XAI evaluation are evaluated in each of the 73 papers in the sample. The most common dimensions are the **contribution of the explanation to human-AI interaction** and the **in-context quality of the explanation**; 54 out of the 73 papers (74%) evaluate one of these dimensions or both of them.

**Table 3:** The dimensions evaluated in each paper in the set.

**Human-AI interaction**	**Human-AI performance**	**In-context expl quality**	***N***	**Papers**
✓			14	Bayer et al., 2022; Branley-Bell et al., 2020; Bunde, 2021; Faulhaber et al., 2021; Fu and Tantithamthavorn, 2022; Kartikeya, 2022; Kühnlenz and Kühnlenz, 2023; Lundberg et al., 2022; Okumura and Nagao, 2023; Reeder et al., 2023; Selten et al., 2023; Upasane et al., 2024; Wang and Yin, 2021; Wysocki et al., 2023
	✓		3	La Gatta et al., 2021a,b; Nazaretsky et al., 2022
		✓	12	Brdnik et al., 2023; Förster et al., 2021; Kim et al., 2023; Meas et al., 2022; Nagy and Molontay, 2023; Polley et al., 2021; Scheers and De Laet, 2021; Schulze-Weddige and Zylowski, 2021; Swamy et al., 2023; van der Waa et al., 2020; Žlahtič et al., 2023; Xu et al., 2023
✓	✓		6	Alufaisan et al., 2021; Cau et al., 2023; Conati et al., 2021; Ghai et al., 2021; Naiseh et al., 2023; Wang et al., 2022a
	✓	✓	3	Eriksson and Grov, 2022; Maltbie et al., 2021; Wang et al., 2022b
✓		✓	28	Abdul et al., 2020; Adhikari et al., 2019; Aechtner et al., 2022; Anjara et al., 2023; Avetisyan et al., 2022; Ben David et al., 2021; Bertrand et al., 2023; Bhattacharya et al., 2023; Chien et al., 2022; Conijn et al., 2023; Das et al., 2023; Deo and Sontakke, 2021; Fernandes et al., 2023; Guo et al., 2022; Hernandez-Bocanegra and Ziegler, 2023; Jang et al., 2023; Khodabandehloo et al., 2021; Larasati, 2022; Moradi and Samwald, 2021; Neves et al., 2021; Ooge et al., 2022; Panigutti et al., 2022, 2023; Schellingerhout et al., 2022; Veldhuis et al., 2022; Warren et al., 2022; Weitz et al., 2021; Zöller et al., 2023
✓	✓	✓	7	Bansal et al., 2021; Buçinca et al., 2020; Confalonieri et al., 2021; Ibrahim et al., 2023; Jmoona et al., 2023; Raab et al., 2023; Schrills and Franke, 2023

**Figure 7:**
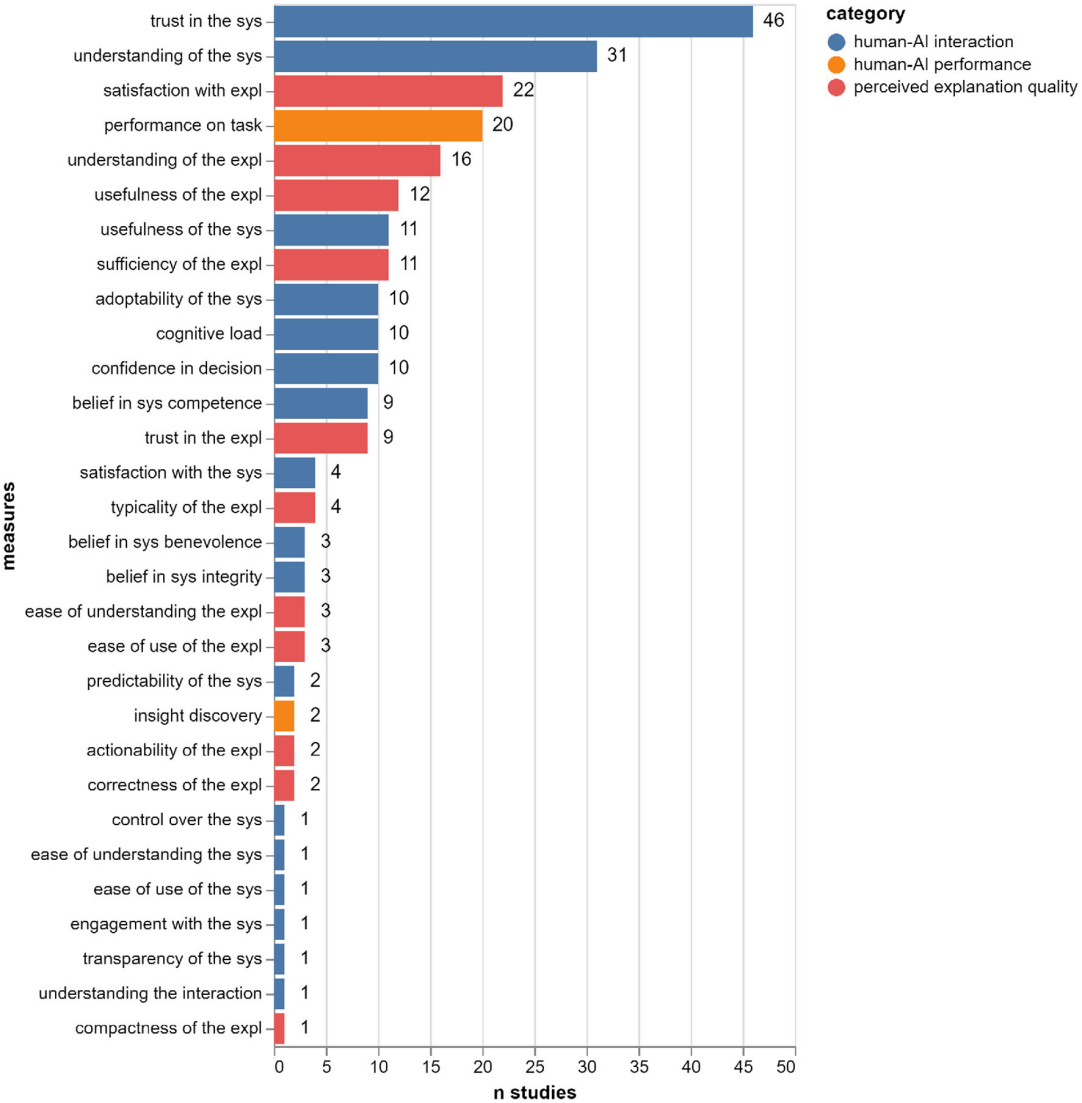
The number of studies in which each meaningfulness component is evaluated (total: 77 studies in 73 papers).

Zooming in on the evaluated properties, Figure 7 illustrates the 30 components of meaningfulness and the number of studies that evaluated each property. The total number of studies reported in our set is 77 since some papers report more than one user study. We can see that the two most common properties are: *trust in the AI system* (used in 46 out of 77 studies) and *understanding of the AI system* (used in 31 out of 77 studies). This could be related to the fact that we used the keywords trust* and understandable in our search query (see Figure 7), thus skewing the sample toward studies that measure these properties (as mentioned in Section 3.1, these words, together with meaningful and interpretable were used in order to focus the search on human-centered evaluation, thus removing them from the search string was not possible). The third most common property is *satisfaction with the explanation* (used in 22 out of 77 studies), and the fourth is *performance on task* (used in 20 out of 77 studies).

#### 4.2.3 Mapping our taxonomy to existing frameworks

As discussed in Section 2, the high-level distinction of existing XAI taxonomies is *evaluation with users* vs. *evaluation without users*. Further categorization usually revolves around either the type of user involved (e.g. lay people vs. domain experts in Doshi-Velez and Kim, 2018) or the type of metric used in the evaluation (quantitative vs. qualitative, objective vs. subjective). In addition to these high-level categories, some of the taxonomies further go into specific evaluated properties (also called *evaluation measures*), similar to our 30 components of meaningfulness. In this section, we show how our taxonomy and the 30 properties we identified relate to four existing taxonomies: Hoffman et al. (2018), Mohseni et al. (2021), Lopes et al. (2022), and Nauta et al. (2023).

**Figure 8:**
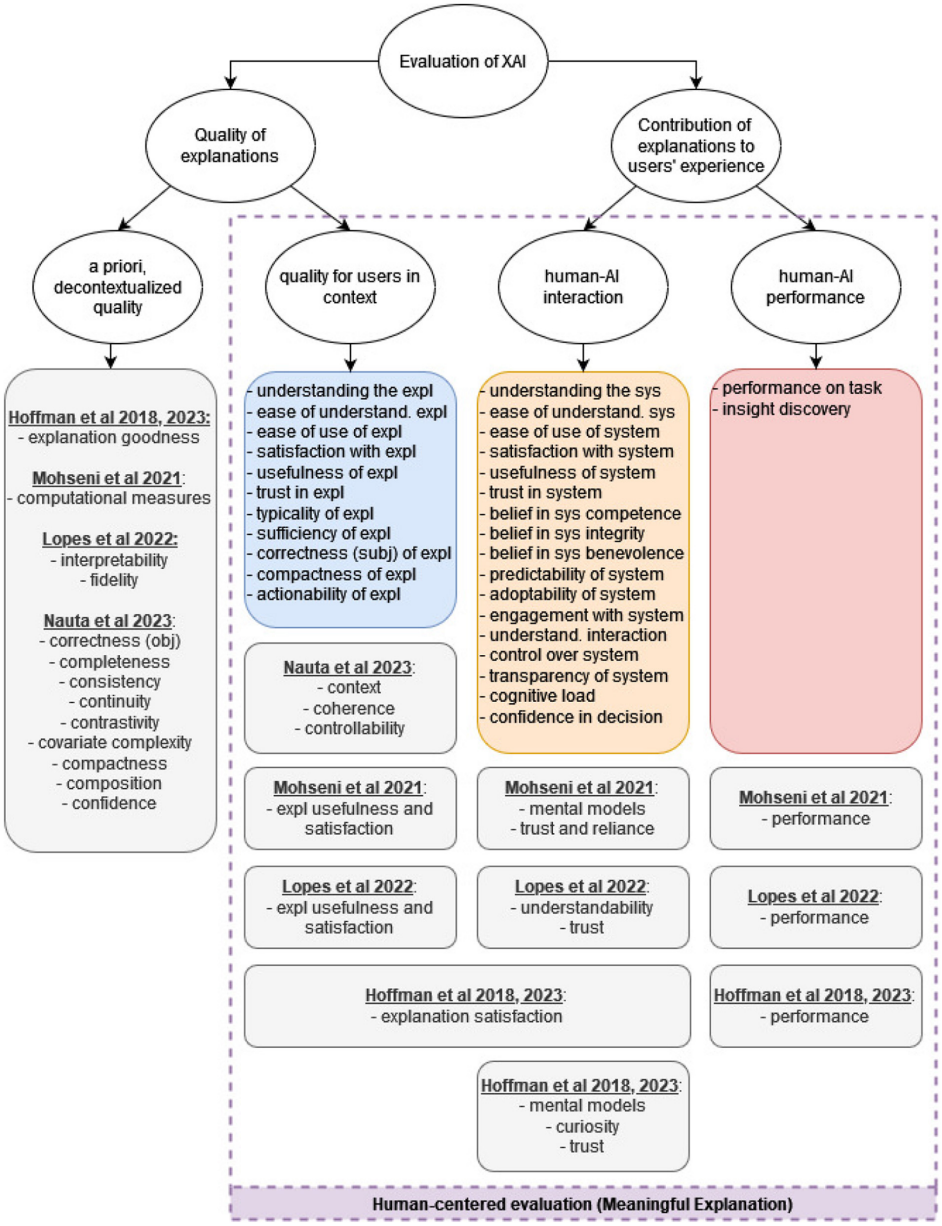
Our taxonomy, compared to other XAI evaluation frameworks. The main difference is in the level of detail of the evaluated properties, and in the novel categorization into the three dimensions of human-centered evaluation.

The hierarchical structure of our taxonomy focuses on **what** is evaluated (which aspects of explanation quality) rather than on **how** it is evaluated (objective vs. subjective, qualitative vs. quantitative). This means that it is possible to map the *what*-elements, i.e. the evaluation measures, of other taxonomies into our structure, and compare them to the 30 properties that we identified. This mapping is shown in Figure 8 and Table 4.

**Table 4:** Mapping of our taxonomy to other XAI evaluation frameworks.

**Our properties**	**Nauta et al. (2023)**	**Mohseni et al. (2021)**	**Lopes et al. (2022)**	**Hoffman et al. (2018, 2023)**
–	Controllability	Explanation usefulness and satisfaction	Explanation usefulness and satisfaction	Explanation satisfaction
Typicality of the explanation Correctness of the explanation (subj)	Coherence			
Usefulness of the explanation Sufficiency of the explanation Compactness of the explanation Actionability of the explanation Satisfaction with the explanation Understanding the explanation Ease of understanding the explanation Ease of use of the explanation Trust in the explanation	Context			
Satisfaction with the system Transparency of the system Usefulness of the system Control over the system Understanding the interaction Ease of use of the system Ease of understanding the system Cognitive load Confidence in decision	–	–	–	
Understanding the system	–	Mental models	Understandability	Mental models
Engagement with the system	–	–	–	Curiosity
Belief in system competence Belief in system integrity Belief in system benevolence Adoptability of the system Predictability of the system Trust in the system	–	Trust and reliance	Trust	Trust
Performance on task Insight discovery	–	Performance	Performance	Performance

#### 4.2.4 A priori explanation quality

As mentioned above, we make a distinction between the a priori, decontextualized aspects of explanation quality, and the in-context explanation quality that is experienced by the user. Our literature review focuses on the latter, therefore we did not map any properties into the *a priori explanation quality* category. Figure 8 shows which properties from other taxonomies can be mapped to this category.

Hoffman et al. (2018, 2023) call this aspect of explanation quality *explanation goodness* and attribute it to the perspective of the system developer, who needs to evaluate explanations before making them available to users. In their framework, this aspect is not necessarily computer-centered or objective; their *explanation goodness checklist* includes subjective evaluation measures, like the explanation's trustworthiness, understandability, sufficiency, etc. What makes it *a priori* is that the judgment is done by the developers, decontextualized from the intended use. In Mohseni et al. (2021), this aspect is called *computational measures* and it also refers to the set of checks performed by the system's developers. It includes both objective and subjective methods that are meant to evaluate the explanation's fidelity to the black-box model (correctness, consistency), and the reliability of the model itself (the training quality).

Lopes et al. (2022) differentiate between *human-centered* and *computer-centered* measures; the latter is a non-human assessment that focuses on two properties: *interpretability* and *fidelity*. While fidelity is objective in nature, interpretability is an inherently subjective property. However, in their framework it is measured quantitatively without humans through various proxies; for example, simplicity and broadness of an explanation are considered components of interpretability, and can be quantitatively measured by a complexity metric (Nguyen and Mart́ınez, 2020).

In the framework of Nauta et al. (2023), the a priori explanation quality can be mapped to nine properties: *correctness, completeness, consistency, continuity, contrastivity, covariate complexity, compactness, composition, confidence*. These properties are components of the objective quality of explanations, decontextualized from the intended use.

#### 4.2.5 In-context explanation quality

The second class of explanation quality measures is the contextualized quality that users attribute to explanations. In our framework, this includes 11 evaluated properties, as shown in Table 4 (blue cells). These properties are mostly, but not exclusively, evaluated subjectively; users are asked about their *satisfaction with the explanation*, the *usefulness of the explanation*, its *typicality*, etc. However, some of the properties can be evaluated objectively as well; for example, *understanding of the explanation* is sometimes evaluated by asking the user to predict or to recall the explainer's output, or by asking the user to answer questions about the explanation.

The other XAI evaluation frameworks also include this aspect of explanation quality, but in a less detailed manner. Mohseni et al. (2021) and Lopes et al. (2022) refer to this aspect as *explanation usefulness and satisfaction*, which is an umbrella term for “*different subjective and objective measures for understandability, usefulness, and sufficiency of details to assess explanatory value for users”* (Mohseni et al., 2021). As shown in Table 4, we map *explanation usefulness and satisfaction* to our 11 components of in-context explanation quality.

Hoffman et al. (2018, 2023) use an even broader category; they have a measure called *explanation satisfaction*, which they define as “*the degree to which users feel that they sufficiently understand the AI system or process being explained to them”*. We interpret this definition as covering all the aspects of our in-context explanation quality, as well as some aspects of explanations' contribution to human-AI interaction, such as the *satisfaction with the AI system, its perceived usefulness*, etc. The exact mapped properties are shown in Table 4.

In Nauta et al. (2023), the in-context quality of explanations can be mapped to three properties: *controllability, coherence*, and *context*. *Coherence* relates to the explanation's plausibility or reasonableness to users, and thus can be mapped to our *typicality of the explanation* and *perceived correctness of the explanation*. *Context* is about the relevance of the explanation to the user and their needs; we map it to nine properties, including the *usefulness of the explanation, it sufficiency, actionability*, etc. (see Table 4). *Controllability* describes how interactive or controllable an explanation is for a user. In our set of papers, the controllability of explanations was not evaluated; we only found studies where the controllability of the AI system was evaluated or where the interactiveness of explanations was the independent variable.

#### 4.2.6 Contribution of explanations to human-AI interaction

Our taxonomy includes 17 properties related to the contribution of explanations to human-AI interaction. As mentioned in Section 4.2.2, the most common properties evaluated in the included papers are *trust in the AI system* and *understanding of the AI system*. These two properties can be also found in the other frameworks that we compare to (except for Nauta et al., 2023's, since they focus on the quality of the explanation itself and do not cover measures related to the user's interaction with the AI system).

The *trust* measure of other frameworks can be mapped to 6 properties in our taxonomy, as shown in Table 4. In some included papers, trust is measured with a single explicit question (e.g. *I trust this AI system to assess the fat content of food*), or with a single objective measure (e.g. percentage of user's agreement with the AI recommendation); in this case, the evaluated property is *trust in the AI system*. Other papers adopt McKnight et al. (2002)'s framework of trusting beliefs and trusting intentions; in this framework, trust is viewed as a multidimensional construct and therefore is evaluated with four properties: *belief in the system's competence, belief in the system's benevolence, belief in the system's integrity* and *adoptability of the system*. Other papers focus on the *predictability of the system* as a measure of trust. In our analysis, we attempt to reflect the evaluation methodologies in the literature as rigorously as possible; therefore, we preserved the variations and ended with six properties to evaluate trust in the system.

The *mental models* or *understandability* measure in other frameworks refers to users' understanding of the AI system. It can be measured both objectively (for example by asking users to predict the model's output and calculating their accuracy), or subjectively (for example with self-ratings). This can be mapped straightforwardly to our *understanding of the AI system* property.

Hoffman et al. (2018, 2023) have an additional measure in their taxonomy, called *curiosity*. They argue that it is important to measure curiosity in the context of XAI because explanations can both promote curiosity (thus setting the stage for the achievement of insights and the development of better mental models) and suppress curiosity (thus reinforcing flawed mental models). Curiosity can be measured by asking the users what were their triggers for asking for explanations, for example *I want to know what the AI just did* or *I want to know what the AI will do next*. This measure can be mapped to our *engagement with the system*, which was evaluated only in one study in our set of papers.

In addition to the properties discussed above, our taxonomy has additional components under the contribution of explanations to human-AI interaction; for example, *satisfaction with the system, controllability of the system, user's cognitive load, user's confidence in the decision*, etc. These properties cannot be mapped to any measures in Mohseni et al. (2021) and Lopes et al. (2022). In Hoffman et al. (2018, 2023), they can be viewed as part of the very broad category of *explanation satisfaction*.

#### 4.2.7 Contribution of explanations to human-AI performance

The third aspect of explanation quality relates to the contribution of explanations to human-AI performance. We identified two properties that relate to this aspects: *performance on task* and *insight discovery*; both can be measured either objectively or subjectively. These two properties can be mapped to the *performance* measure in the other frameworks.

### 4.3 Evaluation procedures for the components of meaningfulness

The previous section focused on *what* components of meaningfulness are evaluated in user studies. In this section, the focus switches to *how* the components of meaningfulness are evaluated.

The analyzed evaluation methodology is characterized mainly by its lack of standardization. We identified nine evaluation frameworks that were applied by more than one study in our set of papers, as shown in Table 5. Out of the 73 papers, only 19 (26%) applied one of these evaluation frameworks; the remaining studies created their own questionnaires and measures, or adapted them from another study that appeared only once in the sample.

**Table 5:** Evaluation frameworks used in the papers.

**Evaluation framework**	**Scale/index**	**Used in**
Hoffman et al. (2018)	Explanation satisfaction scale	Avetisyan et al., 2022; Brdnik et al., 2023; Schrills and Franke, 2023; Warren et al., 2022
McKnight et al. (2002)	Trusting beliefs scale; trusting intentions scale	Bayer et al., 2022; Bertrand et al., 2023; Ghai et al., 2021; Hernandez-Bocanegra and Ziegler, 2023; Ooge et al., 2022
Jian et al. (2000)	Trust in automation scale	Brdnik et al., 2023; Conijn et al., 2023; Kartikeya, 2022; Weitz et al., 2021
Körber (2019)	Trust in automation scale	Faulhaber et al., 2021; Ghai et al., 2021
Hart and Staveland (1988)	Task load index (NASA-TLX)	Bertrand et al., 2023; Chien et al., 2022; Ghai et al., 2021; Schrills and Franke, 2023
Venkatesh et al. (2003), Venkatesh and Bala (2008)	Technology acceptance scale	Bunde, 2021; Panigutti et al., 2022, 2023
O'Brien and Cairns (2015), O'Brien et al. (2018)	User engagement scale	Bertrand et al., 2023; Ghai et al., 2021
Knijnenburg et al. (2012)	User experience scale	Guo et al., 2022; Hernandez-Bocanegra and Ziegler, 2023
Brooke (1996)	System usability scale	Brdnik et al., 2023; Scheers and De Laet, 2021

Moreover, even when the same framework is used, there is variation across the studies in how it is applied. For example, four studies in our sample use the *explanation satisfaction scale* of Hoffman et al. (2018). This scale includes nine items that ask about: understanding the system, satisfaction with the explanation, sufficiency of the explanation, completeness of the explanation, usefulness of the explanation, and transparency of the system; the user needs to rate the importance of each item on a 3-point scale (the revised version of the scale (Hoffman et al., 2023) includes a subset of seven items and a 5-point scale, but the studies in our sample predate its publication). However, none of the four studies in our sample uses the scale in its original form. Brdnik et al. (2023) and Warren et al. (2022) use eight out of the nine items and a 5-point scale. Avetisyan et al. (2022) use only five out of the original nine items and a 7-point scale. Schrills and Franke (2023) adopted the scale as well, but do not provide details about which items and which ratings system they used.

Another example is McKnight et al. (2002)'s framework for measuring trust. The framework was originally developed to measure trust in online vendors, but is used in our sample more broadly to measure trust in AI systems. It includes two scales (the original framework includes four constructs, but only the two identified in our sample are discussed here):

- **Trusting beliefs**: *belief in the system's competence* (four items), *belief in the system's benevolence* (three items) and *belief in the system's integrity* (four items).
- **Trusting intentions**: willingness to depend on the system (fou items), to follow its advice (six items), to give it more information (three items), and to make purchases (three items). The *trusting intentions* construct is called *adoptability of the system* in our framework.

Bayer et al. (2022) use the trusting beliefs scales in their original form, with a 7-point scale; for trusting intentions, they do not use the scales of McKnight et al. (2002), but rather adopt a revised and shorter version of them from Li et al. (2008). Hernandez-Bocanegra and Ziegler (2023) use one item from the *benevolence* scale (out of the original three), and one item from the *competence* scale (out of the original four), with a 5-point scale; they also say that they adopt items from the trusting intentions scale, but we were not able to identify these items in their provided material. Bertrand et al. (2023) use two items from the *benevolence* scale (out of the original three) and three items from the *competence* scale (out of the original four); for some of the items the phrasings from Benbasat and Wang (2005) are used instead of the original phrasings from McKnight et al. (2002) (e.g. “*…wants to understand my needs and preferences”* rather than “*…would do its best to help me”*). Ooge et al. (2022) also uses the phrasings of Benbasat and Wang (2005): four items for competence, three for benevolence, and three for integrity. Ghai et al. (2021) use only one item from each of the trusting beliefs scales not specifying which, and four items for trusting intentions, again not specifying which.

To conclude, the research community does not currently have a unified, standardized approach for XAI evaluation with users. Although standard scales and indices exist for some of the evaluated components (such as trust, explanation satisfaction, cognitive load, engagement), they are not used in the majority of studies. Moreover, even when these scales or indices are used, they are not applied in the original form. This results in large variations in the evaluation methodology across studies, both in what is measured and how it is measured.

## 5 Discussion

The demand for *explainability* of AI systems is becoming a crucial requirement in many domains, both due to legislative requirements and to the realization that successful integration of AI into a decision-making process requires that its end-users understand it, trust it, and perceive it as useful. Despite the growing recognition that explainability serves a user need, and that its effectiveness is closely tied to users' perception, there is no consensus in the research community as to whether evaluation with users is a crucial component of XAI evaluation, and if so, what exactly needs to be evaluated with users and how.

This systematic literature review aimed to address this gap by inventorizing existing work on human-centered XAI evaluation, and organizing it into a detailed taxonomy. The research question we answered is “*How is the meaningfulness of XAI explanations evaluated in user studies?”*. Based on analysis of 73 papers, describing 77 user studies, from empirical XAI literature, we identified 30 components of meaningfulness that can be evaluated with users, and arranged these components into a taxonomy of human-centered XAI evaluation. We propose a novel categorization of evaluation measures, which involves three aspects of meaningfulness:

- The in-context quality of the explanation (11 components). Is the explanation *satisfying, understandable, useful, actionable, sufficient, compact, trustworthy, correct, typical, easy to understand*, and *easy to use*?
- The contribution of the explanation to human-AI interaction (17 components). Does the explanation help the user to better *understand the AI system*? Does it improve the user's perception of the AI system as *trustworthy, useful, satisfying, competent, honest, benevolent, controllable, predictable, transparent, easy to understand, easy to use*, and *engaging*? Does the explanation help the user to better *understand the interaction with the AI system*? Does it make the interaction less *cognitively demanding*? Does it increase the user's *confidence in the decision*? Does it increase the *readiness to adopt the AI system* and use it?
- The contribution of the explanation to human-AI performance (two components). Does the explanation improve the user's *performance on the task*? Does it help the user to *discover new insights*?

Our taxonomy presents a detailed overview of what is currently being evaluated in XAI user studies across multiple domains. It provides a high level of granularity, thus making more explicit the components behind more general umbrella terms like *context* (Nauta et al., 2023), *usefulness* (Mohseni et al., 2021; Lopes et al., 2022) and *satisfaction* (Hoffman et al., 2018, 2023). Moreover, the novel categorization into the above three dimensions highlights that meaningful explainability hinges not only on the quality of the explanation itself, but also on the role it plays in human-AI interaction and human-AI performance. This takeaway contributes toward a more unified research space for XAI evaluation, where insights from machine learning, human-computer interaction, and cognitive sciences can interact and enrich each other.

Our findings also draw attention to the fact that the methodology for human-centered XAI evaluation still faces lack of consensus regarding the measures and metrics to apply, as well as lack of standardized evaluation procedures. This was previously observed and discussed by, among others, Lopes et al. (2022); our review confirms that the situation still persists. This lack of standardization makes it difficult to compare between studies and potentially recognize insights and patterns beyond specific use cases. Moreover, it makes it difficult to arrive at evidence-based XAI guidelines about what constitutes a meaningful explanation for end-users.

For example, a general question one might raise when starting to explore XAI is whether there is clear evidence that providing explanations is beneficial compared to not providing explanations at all. To answer this, we analyzed all the studies in our set which compare between these two conditions (explanation vs. no explanation). Some studies found no added value in providing explanations (e.g., Alufaisan et al., 2021); some studies found that providing explanations is beneficial (e.g., Bunde, 2021); some studies found that providing explanations is beneficial in respect to certain evaluation measures but not others (e.g., Faulhaber et al., 2021); some studies found that providing explanations is beneficial for certain groups of users but not others (e.g., Bayer et al., 2022); some studies found that certain types or formats of explanations are beneficial, but not others (e.g., Avetisyan et al., 2022).

Clearly, there is no straightforward answer to whether providing explanations is generally beneficial, compared to not providing explanations at all. However, there might be insights hidden in these results that currently remain undiscovered because the findings are not mapped into one taxonomy. To take an hypothetical, but very realistic example: consider two studies which evaluate a neural network based AI application that provides a recommendation, accompanied by a feature importance explanation generated by a standard XAI method like SHAP. Both studies evaluate whether providing this type of explanation improves users' trust in the AI system and their intention to adopt the system for decision support. Both studies apply a between-subject design, and compare between a condition where an explanation is provided and a condition where no explanation is provided. One study finds that providing explanations significantly improves users' trust in the AI system and their intention to adopt it; the other study finds no significant difference between the conditions. In the current state of affairs of XAI evaluation, these results remain disconnected from each other, since it is very likely that the two studies used different definitions of trust and adoptability, and different methodologies of how to evaluate these constructs with users.

However, if they were to use a standard taxonomy of evaluation measures and a standard evaluation procedure, these results could have led to a deeper level in the exploration of explainability. If we controlled for methodology, we could ask what are the *real-world* differences between the two use cases, such as the characteristics of the users, the application domain, the decision-making process in which the AI is integrated, etc. If the difference in the results stems from real important variations between the use cases, rather than from methodological inconsistency, it can teach us what contextual aspects affect explainability needs and explainability effectiveness.

There is reason to believe that such contextual aspects are very important, if we want to design XAI systems that truly address user needs and achieve successful and meaningful human-AI collaboration. For example, studies show that explanation needs and the ability to benefit from explanations vary according to user characteristics, such as their level of AI expertise (e.g., Ghai et al., 2021), domain expertise (e.g., Bayer et al., 2022), and personal traits like the *need for cognition*^5^ (e.g., Conati et al., 2021), or personal decision-making style (e.g., Hernandez-Bocanegra and Ziegler, 2023).

In addition to user characteristics, the particularities of the specific decision-making process in which the AI system is embedded can also affect explainability needs. In a recent study, Kim et al. (in press) analyzed interviews with stakeholders from two use cases in the financial sector, in which a decision-support XAI system is already in use. The first use case concerns credit approval; the AI system outputs a score which indicates the chance of a credit request to be approved. The second use case concerns fraud detection; the AI system outputs a score which indicates the risk of a claim to be fraudulent. In both use cases, a local feature importance explanation is provided, which shows the most important features contributing to the score. Despite similarities between the use cases in terms of the domain (finance), the type of users (non-technical domain experts), the system's output (score) and the explanation type (local feature importance), a difference in explainability needs was identified. Interviewees in the fraud detection use case indicated that feature importance explanations are not sufficient for their needs, since fraud is not about the individual factors by themselves, but rather about the ability to combine the factors into a plausible fraud scenario. In this specific decision-making process, seeing the explanation as separate factors is not meaningful, because the overall narrative is lost. In the credit approval use case, on the other hand, this need for a coherent narrative was not mentioned, probably because the process of approving a credit request is aligned with checking whether individual factors are satisfied.

These findings suggest that explainability is sensitive to contextual factors, such as the characteristics of the intended users and the particularities of the decision-making process in which the XAI system is embedded. The field of human-centered XAI evaluation can therefore benefit from a more systematic and comprehensive exploration of how these contextual factors affect explainability needs and explainability effectiveness. To achieve this, more standardization is needed in respect to the applied evaluation measures (what to evaluate) and procedures (how to evaluate). By mapping in detail the current state of affairs, this literature review can serve as a step toward this goal.

Our taxonomy (Section 4.2) presents all the diverse and partially overlapping components of a meaningful explanation that are currently evaluated in XAI user studies (what is evaluated); our overview of frameworks (Section 4.3) shows the evaluation procedures that are currently applied in more than one study (how it is evaluated). Building on these resources, future work can focus on additional standardization efforts for each of the identified properties. This involves, first of all, providing precise definitions for complex constructs, such as *understanding of the AI system*, so that future studies can use consistent terminology. Following this, a consensus on standardized measurement methods for each property should be established; for example, which objective and subjective metrics to apply in order to evaluate understanding of the AI system. Finally, the agreed-upon measures and metrics can be compiled into evaluation procedures; for example, a standard questionnaire for subjective evaluation of the understanding of the AI system. We believe that this way forward is necessary in order to further promote comparability of XAI methods across different studies, and gain insights that go beyond individual use cases.

### 5.1 Limitations

This systematic review provides a detailed overview of human-centered XAI evaluation measures. Our review includes only XAI evaluation studies that are conducted with users. For a recent comprehensive review of computer-centered XAI evaluation measures, the reader is referred to Nauta et al. (2023).

Moreover, we focus on a subset of AI systems. The review does not include papers discussing agents (robots or chatbots) which explain their actions or their failures to users; rather, our focus is on decision-support AI systems which explain either the model itself (training data, training procedure, performance metrics), or the model's outputs (predictions, recommendations). Within these AI-based decision-support systems, we do not include computer vision systems, which are trained on image data and are typically characterized by image-based explanations (e.g., heatmaps).

The proposed taxonomy presents the evaluation measures that we encountered in the reviewed user studies. We minimized our own interpretation and showed the diversity as it manifests in the literature. This means that some of the properties in the taxonomy (partially) overlap.

With regard to operationalization of the surveyed measures and procedures, this work provides an inventory of available methods and standard frameworks that researchers can choose from. However, we do not provide recommendations as to which methods are most suitable for specific scenarios or use cases. This type of mapping is currently difficult to discern from the existing literature and is therefore left for future work.

## 6 Conclusions and future work

We performed a systematic literature review of 73 papers that evaluate XAI systems with users, focusing specifically on systems that are based on tabular or textual input data. We found that there are many different properties which are considered by XAI researchers as important components of what makes explanations meaningful to users. We proposed to categorize these components in a taxonomy along three dimensions: the contextualized quality of the explanation, the contribution of the explanation to human-AI interaction, and the contribution of the explanation to human-AI performance. Our taxonomy makes the main aspects of human-centered explanation quality explicit. In future work, additional evaluation measures can be added to the existing categories to extend the taxonomy further; alternatively, some partially overlapping measures can be condensed and standardized. In addition, future work should explore whether the taxonomy can be applied to XAI systems not covered by the current review, i.e. autonomous agents and computer vision systems.

In our view, the next step in the exploration of human-centered XAI is understanding the real-world differences that affect explainability needs and explainability effectiveness across use cases. This includes contextual aspects such as user characteristics, the application domain, and the decision-making process in which the AI system is embedded. To be able to investigate these questions and advance the field, two things are needed. First, the evaluation methodology of user studies needs to be standardized, to facilitate meaningful comparison across studies and discovery of insights beyond specific use cases. Second, user studies need to be conducted in an application-grounded setup, i.e., with a real task and the intended end-users as participants.

## Data Availability

The original contributions presented in the study are included in the article/Supplementary material, further inquiries can be directed to the corresponding author.

## Footnotes

^1^There is no consensus regarding the exact components of this decontextualized “explanation goodness”; the crucial point is that whether an explanation is “good” is determined without involving the users of the AI system.

^2^Note that in computer-centered evaluation (which is the focus of Nauta et al., 2023), quantitative metrics are necessarily objective, but in human-centered evaluation, quantitative metrics can be both objective (e.g. time spent on task) and subjective (e.g. ratings).

^3^Examples of common playground applications are house price predictions based on the Boston Housing dataset, recidivism prediction based on the COMPAS dataset, or income prediction based on the Census Income dataset.

^4^It should be noted that the terminology differs from paper to paper; for example, Laato et al. (2022) uses *end-users* to refer both to the people who use the AI system and to the people affected by AI-based decisions, but this is not the definition adopted here.

^5^Need for cognition (Cacioppo and Petty, 1982) is defined as the individual tendency to engage in effortful cognitive activities.

## References

[B1] Abdul A. von der Weth C. Kankanhalli M. Lim B. Y. (2020). “COGAM: measuring and moderating cognitive load in machine learning model explanations,” in Proceedings of the 2020 CHI Conference on Human Factors in Computing Systems (New York, NY: ACM), 1–14. 10.1145/3313831.3376615

[B2] Adhikari A. Tax D. M. Satta R. Faeth M. (2019). “LEAFAGE: example-based and feature importance-based explanations for black-box ML models,” in 2019 IEEE international conference on fuzzy systems (FUZZ-IEEE) (New Orleans, LA: IEEE), 1–7. 10.1109/FUZZ-IEEE.2019.8858846

[B3] Aechtner J. Cabrera L. Katwal D. Onghena P. Valenzuela D. P. Wilbik A. . (2022). “Comparing user perception of explanations developed with XAI methods,” in 2022 IEEE International Conference on Fuzzy Systems (FUZZ-IEEE) (Padua: IEEE), 1–7. 10.1109/FUZZ-IEEE55066.2022.9882743

[B4] Alufaisan Y. Marusich L. R. Bakdash J. Z. Zhou Y. Kantarcioglu M. (2021). Does explainable artificial intelligence improve human decision-making? Proc. AAAI Conf. Artif. Intell. 35, 6618–6626. 10.1609/aaai.v35i8.16819

[B5] Anjara S. G. Janik A. Dunford-Stenger A. Mc Kenzie K. Collazo-Lorduy A. Torrente M. . (2023). Examining explainable clinical decision support systems with think aloud protocols. PLoS ONE 18:e0291443. 10.1371/journal.pone.0291443 37708135 PMC10501571

[B6] Anjomshoae S. Najjar A. Calvaresi D. Främling K. (2019). “Explainable agents and robots: results from a systematic literature review,”' in 18th International Conference on Autonomous Agents and Multiagent Systems (AAMAS 2019), Montreal, Canada, May 13–17 (Montreal, QC: International Foundation for Autonomous Agents and Multiagent Systems), 1078–1088.

[B7] Antoniadi A. M. Du Y. Guendouz Y. Wei L. Mazo C. Becker B. A. . (2021). Current challenges and future opportunities for XAI in machine learning-based clinical decision support systems: a systematic review. Appl. Sci. 11:5088. 10.3390/app11115088

[B8] Avetisyan L. Ayoub J. Zhou F. (2022). Investigating explanations in conditional and highly automated driving: the effects of situation awareness and modality. Transp. Res. F: Traffic Psychol. Behav. 89, 456–466. 10.1016/j.trf.2022.07.010

[B9] Bansal G. Wu T. Zhou J. Fok R. Nushi B. Kamar E. . (2021). “Does the whole exceed its parts? The effect of AI explanations on complementary team performance,” in Proceedings of the 2021 CHI Conference on Human Factors in Computing Systems (New York, NY: ACM), 1–16. 10.1145/3411764.3445717

[B10] Bayer S. Gimpel H. Markgraf M. (2022). The role of domain expertise in trusting and following explainable AI decision support systems. J. Decis. Syst. 32, 110–138. 10.1080/12460125.2021.1958505

[B11] Ben David D. Resheff Y. S. Tron T. (2021). “Explainable AI and adoption of financial algorithmic advisors: an experimental study,” in Proceedings of the 2021 AAAI/ACM Conference on AI, Ethics, and Society (New York, NY: ACM), 390–400. 10.1145/3461702.3462565

[B12] Benbasat I. Wang W. (2005). Trust in and adoption of online recommendation agents. J. Assoc. Inf. Syst. 6:4. 10.17705/1jais.00065

[B13] Bertrand A. Belloum R. Eagan J. R. Maxwell W. (2022). “How cognitive biases affect XAI-assisted decision-making: a systematic review,” in Proceedings of the 2022 AAAI/ACM Conference on AI, Ethics, and Society, 78–91. 10.1145/3514094.3534164

[B14] Bertrand A. Eagan J. R. Maxwell W. (2023). “Questioning the ability of feature-based explanations to empower non-experts in robo-advised financial decision-making,” in Proceedings of the 2023 ACM Conference on Fairness, Accountability, and Transparency (New York, NY: ACM), 943–958. 10.1145/3593013.3594053

[B15] Bhattacharya A. Ooge J. Stiglic G. Verbert K. (2023). “Directive explanations for monitoring the risk of diabetes onset: introducing directive data-centric explanations and combinations to support what-if explorations,” in Proceedings of the 28th International Conference on Intelligent User Interfaces (New York, NY: ACM), 204–219. 10.1145/3581641.3584075

[B16] Borrego-Díaz J. Galán-Páez J. (2022). Explainable artificial intelligence in data science: from foundational issues towards socio-technical considerations. Minds Mach. 32, 485–531. 10.1007/s11023-022-09603-z

[B17] Branley-Bell D. Whitworth R. Coventry L. (2020). “User trust and understanding of explainable AI: exploring algorithm visualisations and user biases,” in International Conference on Human-Computer Interaction (Cham: Springer), 382–399. 10.1007/978-3-030-49065-2_27

[B18] Brdnik S. Podgorelec V. Šumak B. (2023). Assessing perceived trust and satisfaction with multiple explanation techniques in XAI-enhanced learning analytics. Electronics 12, 2594. 10.3390/electronics12122594

[B19] Bright T. J. Wong A. Dhurjati R. Bristow E. Bastian L. Coeytaux R. R. . (2012). Effect of clinical decision-support systems: a systematic review. Ann. Intern. Med. 157, 29–43. 10.7326/0003-4819-157-1-201207030-00450 22751758

[B20] Brooke J. (1996). “SUS-a quick and dirty usability scale,” in Usability Evaluation in Industry, eds. P. W. Jordan, B. Thomas, B. A. Weerdmeester, and I. L. McClelland (New York, NY: Taylor & Francis), 189–194.

[B21] Buçinca Z. Lin P. Gajos K. Z. Glassman E. L. (2020). “Proxy tasks and subjective measures can be misleading in evaluating explainable AI systems,” in Proceedings of the 25th international conference on intelligent user interfaces (New York, NY: ACM), 454–464. 10.1145/3377325.3377498

[B22] Bunde E. (2021). “AI-assisted and explainable hate speech detection for social media moderators: a design science approach,” in Proceedings of the 54th Hawaii International Conference on System Sciences (Hawaii).

[B23] Cacioppo J. T. Petty R. E. (1982). The need for cognition. J. Pers. Soc. Psychol. 42:116. 10.1037/0022-3514.42.1.116

[B24] Cau F. M. Hauptmann H. Spano L. D. Tintarev N. (2023). “Supporting high-uncertainty decisions through AI and logic-style explanations,” in Proceedings of the 28th International Conference on Intelligent User Interfaces (New York, NY: ACM), 251–263. 10.1145/3581641.3584080

[B25] Chien S.-Y. Yang C.-J. Yu F. (2022). XFlag: explainable fake news detection model on social media. Int. J. Hum. Comput. Interact. 38, 1808–1827. 10.1080/10447318.2022.2062113

[B26] Chromik M. Schuessler M. (2020). “A taxonomy for human subject evaluation of black-box explanations in XAI,” in Proceedings of the Workshop on Explainable Smart Systems for Algorithmic Transparency in Emerging Technologies Co-located with 25th International Conference on Intelligent User Interfaces (IUI 2020), Cagliari, Italy, March 17, 2020, Vol. 2582, eds. A. Smith-Renner, S. Kleanthous, B. Lim, T. Kuflik, S. Stumpf, J. Otterbacher, A. Sarkar, C. Dugan, and A. Shulner Tal (CEUR-WS.org). Available at: http://ceur-ws.org/Vol-2582/paper9.pdf

[B27] Conati C. Barral O. Putnam V. Rieger L. (2021). Toward personalized XAI: a case study in intelligent tutoring systems. Artif. Intell. 298:103503. 10.1016/j.artint.2021.103503

[B28] Confalonieri R. Weyde T. Besold T. R. del Prado Martín F. M. (2021). Using ontologies to enhance human understandability of global *post-hoc* explanations of black-box models. Artif. Intell. 296:103471. 10.1016/j.artint.2021.103471

[B29] Conijn R. Kahr P. Snijders C. (2023). The effects of explanations in automated essay scoring systems on student trust and motivation. J. Learn. Anal. 10, 37–53. 10.18608/jla.2023.7801

[B30] Das D. Nishimura Y. Vivek R. P. Takeda N. Fish S. T. Ploetz T. . (2023). Explainable activity recognition for smart home systems. ACM Trans. Interact. Intell. Syst. 13, 1–39. 10.1145/3561533

[B31] Deo S. Sontakke N. (2021). “User-centric explainability in fintech applications,” in HCI International 2021-Posters: 23rd HCI International Conference, HCII 2021, Virtual Event, July 24-29, 2021, Proceedings, Part II 23 (Cham: Springer), 481–488. 10.1007/978-3-030-78642-7_64

[B32] Diakopoulos N. (2014). Algorithmic accountability reporting: on the investigation of black boxes. Digit. J. 3, 398–415. 10.1080/21670811.2014.976411

[B33] Doshi-Velez F. Kim B. (2018). “Considerations for evaluation and generalization in interpretable machine learning,” in Explainable and interpretable models in computer vision and Machine Learning, eds. H. J. Escalante, S. Escalera, I. Guyon, X. Baró, Y. Güçlütürk, U. Güçlü, et al. (Cham: Springer), 3–17. 10.1007/978-3-319-98131-4_1

[B34] Eriksson H. S. Grov G. (2022). “Towards XAI in the soc-a user centric study of explainable alerts with shap and lime,” in 2022 IEEE International Conference on Big Data (Big Data) (IEEE), 2595–2600. 10.1109/BigData55660.2022.10020248

[B35] Faulhaber A. K. Ni I. Schmidt L. (2021). The effect of explanations on trust in an assistance system for public transport users and the role of the propensity to trust. Proc. Mensch Comput. 2021, 303–310. 10.1145/3473856.3473886

[B36] Fernandes G. J. Choi A. Schauer J. M. Pfammatter A. F. Spring B. J. Darwiche A. . (2023). An explainable artificial intelligence software tool for weight management experts (PRIMO): mixed methods study. J. Med. Internet Res. 25:e42047. 10.2196/42047 37672333 PMC10512114

[B37] Ferreira J. J. Monteiro M. S. (2020). “What are people doing about XAI user experience? A survey on AI explainability research and practice,” in Design, User Experience, and Usability. Design for Contemporary Interactive Environments: 9th International Conference, DUXU 2020, Held as Part of the 22nd HCI International Conference, HCII 2020, Copenhagen, Denmark, July 19-24, 2020, Proceedings, Part II 22 (Cham: Springer), 56–73. 10.1007/978-3-030-49760-6_4

[B38] Förster M. Hühn P. Klier M. Kluge K. (2021). “Capturing users' reality: a novel approach to generate coherent counterfactual explanations,” in Hawaii International Conference on System Sciences. Available at: https://api.semanticscholar.org/CorpusID:232412682

[B39] Fu M. Tantithamthavorn C. (2022). GPT2SP: a transformer-based agile story point estimation approach. IEEE Trans. Softw. Eng. 49, 611–625. 10.1109/TSE.2022.3158252

[B40] Ghai B. Liao Q. V. Zhang Y. Bellamy R. Mueller K. (2021). Explainable active learning (XAL) toward AI explanations as interfaces for machine teachers. Proc. ACM Hum. Comput. Interact. 4(CSCW3), 1–28. 10.1145/3432934

[B41] Guo L. Daly E. M. Alkan O. Mattetti M. Cornec O. Knijnenburg B. . (2022). “Building trust in interactive machine learning via user contributed interpretable rules,” in 27th International Conference on Intelligent User Interfaces (New York, NY: ACM), 537–548. 10.1145/3490099.3511111

[B42] Haque A. B. Islam A. N. Mikalef P. (2023). Explainable artificial intelligence (XAI) from a user perspective: a synthesis of prior literature and problematizing avenues for future research. Technol. Forecast. Soc. Change 186:122120. 10.1016/j.techfore.2022.122120

[B43] Hart S. G. Staveland L. E. (1988). Development of NASA-TLX (Task Load Index): results of empirical and theoretical research. Adv. Psychol. 52, 139–183. 10.1016/S0166-4115(08)62386-9

[B44] Hernandez-Bocanegra D. C. Ziegler J. (2023). Explaining recommendations through conversations: dialog model and the effects of interface type and degree of interactivity. ACM Trans. Interact. Intell. Syst. 13, 1–47. 10.1145/3579541

[B45] Hoffman R. R. Mueller S. T. Klein G. Litman J. (2018). Metrics for explainable AI: challenges and prospects. arXiv [Preprint]. arXiv:1812.04608. 10.48550/arXiv.1812.0460839281476

[B46] Hoffman R. R. Mueller S. T. Klein G. Litman J. (2023). Measures for explainable AI: explanation goodness, user satisfaction, mental models, curiosity, trust, and human-AI performance. Front. Comput. Sci. 5:1096257. 10.3389/fcomp.2023.1096257

[B47] Ibrahim L. Ghassemi M. M. Alhanai T. (2023). “Do explanations improve the quality of AI-assisted human decisions? An algorithm-in-the-loop analysis of factual and counterfactual explanations,” in Proceedings of the 2023 International Conference on Autonomous Agents and Multiagent Systems (London), 326–334.

[B48] Jang J. Kim M. Bui T.-C. Li W.-S. (2023). “Toward interpretable machine learning: Constructing polynomial models based on feature interaction trees,” in Pacific-Asia Conference on Knowledge Discovery and Data Mining (Cham: Springer), 159–170. 10.1007/978-3-031-33377-4_13

[B49] Jian J.-Y. Bisantz A. M. Drury C. G. (2000). Foundations for an empirically determined scale of trust in automated systems. Int. J. Cogn. Ergon. 4, 53–71. 10.1207/S15327566IJCE0401_0427885969

[B50] Jmoona W. Ahmed M. U. Islam M. R. Barua S. Begum S. Ferreira A. . (2023). “Explaining the unexplainable: role of XAI for flight take-off time delay prediction,” in IFIP International Conference on Artificial Intelligence Applications and Innovations (Cham: Springer), 81–93. 10.1007/978-3-031-34107-6_7

[B51] Jung J. Lee H. Jung H. Kim H. (2023). Essential properties and explanation effectiveness of explainable artificial intelligence in healthcare: a systematic review. Heliyon 9:e16110. 10.1016/j.heliyon.2023.e16110 37234618 PMC10205582

[B52] Kartikeya A. (2022). “Examining correlation between trust and transparency with explainable artificial intelligence,” in Science and Information Conference (Cham: Springer), 353–358. 10.1007/978-3-031-10464-0_23

[B53] Khodabandehloo E. Riboni D. Alimohammadi A. (2021). HealthXAI: Collaborative and explainable AI for supporting early diagnosis of cognitive decline. Future Gener. Comput. Syst. 116, 168–189. 10.1016/j.future.2020.10.030

[B54] Kim D. Song Y. Kim S. Lee S. Wu Y. Shin J. . (2023). How should the results of artificial intelligence be explained to users? - Research on consumer preferences in user-centered explainable artificial intelligence. Technol. Forecast. Soc. Change 188:122343. 10.1016/j.techfore.2023.122343

[B55] Kim J. Maathuis H. van Montfort K. Sent D. (in press). “Identifying XAI user needs: gaps between literature use cases in the financial sector,” in Proceedings of the 2nd Workshop on Responsible Applied Artificial Intelligence (RAAIT), at HHAI 2024 (Malmö: CEUR Workshop Proceedings (CEUR-WS.org)). Accepted for publication.

[B56] Knijnenburg B. P. Willemsen M. C. Gantner Z. Soncu H. Newell C. (2012). Explaining the user experience of recommender systems. User Model. User-adapt. Interact. 22, 441–504. 10.1007/s11257-011-9118-4

[B57] Körber M. (2019). “Theoretical considerations and development of a questionnaire to measure trust in automation,” in Proceedings of the 20th Congress of the International Ergonomics Association (IEA 2018) Volume VI: Transport Ergonomics and Human Factors (TEHF), Aerospace Human Factors and Ergonomics 20 (Cham: Springer), 13–30. 10.1007/978-3-319-96074-6_2

[B58] Kühnlenz K. Kühnlenz B. (2023). “Study on the impact of situational explanations and prior information given to users on trust and perceived intelligence in autonomous driving in a video-based 2x2 design,” in 2023 32nd IEEE International Conference on Robot and Human Interactive Communication (RO-MAN) (Busan: IEEE), 1509–1513. 10.1109/RO-MAN57019.2023.10309319

[B59] La Gatta V. Moscato V. Postiglione M. Sperli G. (2021a). CASTLE: cluster-aided space transformation for local explanations. Expert Syst. Appl. 179:115045. 10.1016/j.eswa.2021.115045

[B60] La Gatta V. Moscato V. Postiglione M. Sperlì G. (2021b). PASTLE: pivot-aided space transformation for local explanations. Pattern Recognit. Lett. 149, 67–74. 10.1016/j.patrec.2021.05.018

[B61] Laato S. Tiainen M. Najmul Islam A. Mäntymäki M. (2022). How to explain AI systems to end users: a systematic literature review and research agenda. Internet Res. 32, 1–31. 10.1108/INTR-08-2021-0600

[B62] Larasati R. (2022). “Explainable AI for breast cancer diagnosis: application and user's understandability perception,” in 2022 International Conference on Electrical, Computer and Energy Technologies (ICECET) (Prague: IEEE), 1–6. 10.1109/ICECET55527.2022.9872950

[B63] Li X. Hess T. J. Valacich J. S. (2008). Why do we trust new technology? A study of initial trust formation with organizational information systems. J. Strateg. Inf. Syst. 17, 39–71. 10.1016/j.jsis.2008.01.001

[B64] Liao Q. V. Gruen D. Miller S. (2020). “Questioning the AI: informing design practices for explainable AI user experiences,” in Proceedings of the 2020 CHI conference on human factors in computing systems (New York, NY: ACM), 1–15. 10.1145/3313831.3376590

[B65] Liao Q. V. Varshney K. R. (2021). Human-centered explainable AI (XAI): from algorithms to user experiences. arXiv [Preprint]. arXiv:2110.10790. 10.48550/arXiv.2110.1079037045794

[B66] Linardatos P. Papastefanopoulos V. Kotsiantis S. (2020). Explainable AI: a review of machine learning interpretability methods. Entropy 23:18. 10.3390/e23010018 33375658 PMC7824368

[B67] Lopes P. Silva E. Braga C. Oliveira T. Rosado L. (2022). XAI systems evaluation: a review of human and computer-centred methods. Appl. Sci. 12:9423. 10.3390/app12199423

[B68] Loyola-Gonzalez O. (2019). Black-box vs. white-box: understanding their advantages and weaknesses from a practical point of view. IEEE Access 7, 154096–154113. 10.1109/ACCESS.2019.2949286

[B69] Lundberg H. Mowla N. I. Abedin S. F. Thar K. Mahmood A. Gidlund M. . (2022). Experimental analysis of trustworthy in-vehicle intrusion detection system using eXplainable Artificial Intelligence (XAI). IEEE Access 10, 102831–102841. 10.1109/ACCESS.2022.3208573

[B70] Maltbie N. Niu N. Van Doren M. Johnson R. (2021). “XAI tools in the public sector: a case study on predicting combined sewer overflows,” in Proceedings of the 29th ACM Joint Meeting on European Software Engineering Conference and Symposium on the Foundations of Software Engineering (New York, NY: ACM), 1032–1044. 10.1145/3468264.3468547

[B71] McKnight D. H. Choudhury V. Kacmar C. (2002). Developing and validating trust measures for e-commerce: an integrative typology. Inf. Syst. Res. 13, 334–359. 10.1287/isre.13.3.334.8119642375

[B72] Meas M. Machlev R. Kose A. Tepljakov A. Loo L. Levron Y. . (2022). Explainability and transparency of classifiers for air-handling unit faults using explainable artificial intelligence (XAI). Sensors 22:6338. 10.3390/s22176338 36080795 PMC9460735

[B73] Meske C. Bunde E. Schneider J. Gersch M. (2022). Explainable artificial intelligence: objectives, stakeholders, and future research opportunities. Inf. Syst. Manag. 39, 53–63. 10.1080/10580530.2020.1849465

[B74] Miller T. (2019). Explanation in artificial intelligence: insights from the social sciences. Artif. Intell. 267, 1–38. 10.1016/j.artint.2018.07.007

[B75] Mohseni S. Zarei N. Ragan E. D. (2021). A multidisciplinary survey and framework for design and evaluation of explainable AI systems. ACM Trans. Interact. Intell. Syst. 11, 1–45. 10.1145/3387166

[B76] Moradi M. Samwald M. (2021). *Post-hoc* explanation of black-box classifiers using confident itemsets. Expert Syst. Appl. 165:113941. 10.1016/j.eswa.2020.11394133534720

[B77] Nagy M. Molontay R. (2023). Interpretable dropout prediction: towards XAI-based personalized intervention. Int. J. Artif. Intell. Educ. 34, 274–300. 10.1007/s40593-023-00331-8

[B78] Naiseh M. Al-Thani D. Jiang N. Ali R. (2023). How the different explanation classes impact trust calibration: the case of clinical decision support systems. Int. J. Hum. Comput. Stud. 169:102941. 10.1016/j.ijhcs.2022.102941

[B79] Nauta M. Trienes J. Pathak S. Nguyen E. Peters M. Schmitt Y. . (2023). From anecdotal evidence to quantitative evaluation methods: a systematic review on evaluating explainable AI. ACM Comput. Surveys 55(13s), 1–42. 10.1145/3583558

[B80] Nazaretsky T. Bar C. Walter M. Alexandron G. (2022). “Empowering teachers with AI: co-designing a learning analytics tool for personalized instruction in the science classroom,” in LAK22: 12th International Learning Analytics and Knowledge Conference (New York, NY: ACM), 1–12. 10.1145/3506860.3506861

[B81] Neves I. Folgado D. Santos S. Barandas M. Campagner A. Ronzio L. . (2021). Interpretable heartbeat classification using local model-agnostic explanations on ECGs. Comput. Biol. Med. 133:104393. 10.1016/j.compbiomed.2021.104393 33915362

[B82] Ngai E. W. Hu Y. Wong Y. H. Chen Y. Sun X. (2011). The application of data mining techniques in financial fraud detection: a classification framework and an academic review of literature. Decis. Support Syst. 50, 559–569. 10.1016/j.dss.2010.08.006

[B83] Nguyen A.-p. Martínez M. R. (2020). On quantitative aspects of model interpretability. arXiv [Preprint]. arXiv:2007.07584. 10.48550/arXiv.2007.07584

[B84] O'Brien H. Cairns P. (2015). An empirical evaluation of the User Engagement Scale (UES) in online news environments. Inf. Process. Manag. 51, 413–427. 10.1016/j.ipm.2015.03.003

[B85] O'Brien H. L. Cairns P. Hall M. (2018). A practical approach to measuring user engagement with the refined user engagement scale (UES) and new ues short form. Int. J. Hum. Comput. Stud. 112, 28–39. 10.1016/j.ijhcs.2018.01.004

[B86] Okumura H. Nagao T. (2023). “MIPCE: generating multiple patches counterfactual-changing explanations for time series classification,” in International Conference on Artificial Neural Networks (Cham: Springer), 231–242. 10.1007/978-3-031-44223-0_19

[B87] Ooge J. Kato S. Verbert K. (2022). “Explaining recommendations in e-learning: effects on adolescents' trust,” in 27th International Conference on Intelligent User Interfaces (New York, NY: ACM), 93–105. 10.1145/3490099.3511140

[B88] Panigutti C. Beretta A. Fadda D. Giannotti F. Pedreschi D. Perotti A. . (2023). Co-design of human-centered, explainable AI for clinical decision support. ACM Trans. Interact. Intell. Syst., 13, 1–35. 10.1145/3587271

[B89] Panigutti C. Beretta A. Giannotti F. Pedreschi D. (2022). “Understanding the impact of explanations on advice-taking: a user study for AI-based clinical decision support systems,” in Proceedings of the 2022 CHI Conference on Human Factors in Computing Systems (New York, NY: ACM), 1–9. 10.1145/3491102.3502104

[B90] Polley S. Koparde R. R. Gowri A. B. Perera M. Nuernberger A. (2021). “Towards trustworthiness in the context of explainable search,” in Proceedings of the 44th International ACM SIGIR Conference on Research and Development in Information Retrieval (New York, NY: ACM), 2580–2584. 10.1145/3404835.3462799

[B91] Raab D. Theissler A. Spiliopoulou M. (2023). XAI4EEG: spectral and spatio-temporal explanation of deep learning-based seizure detection in EEG time series. Neural Comput. Appl. 35, 10051–10068. 10.1007/s00521-022-07809-x

[B92] Rai A. (2020). Explainable AI: from black box to glass box. J. Acad. Mark. Sci. 48, 137–141. 10.1007/s11747-019-00710-5

[B93] Reeder S. Jensen J. Ball R. (2023). “Evaluating explainable AI (XAI) in terms of user gender and educational background,” in International Conference on Human-Computer Interaction (Cham: Springer), 286–304. 10.1007/978-3-031-35891-3_18

[B94] Scheers H. De Laet T. (2021). “Interactive and explainable advising dashboard opens the black box of student success prediction,” in Technology-Enhanced Learning for a Free, Safe, and Sustainable World: 16th European Conference on Technology Enhanced Learning, EC-TEL 2021, Bolzano, Italy, September 20-24, 2021, Proceedings 16 (Cham: Springer), 52–66. 10.1007/978-3-030-86436-1_5

[B95] Schellingerhout R. Medentsiy V. Marx M. (2022). Explainable career path predictions using neural models.

[B96] Schrills T. Franke T. (2023). How do users experience traceability of AI systems? Examining subjective information processing awareness in automated insulin delivery (AID) systems. ACM Trans. Interact. Intell. Syst. 13, 1–34. 10.1145/3588594

[B97] Schulze-Weddige S. Zylowski T. (2021). “User study on the effects explainable AI visualizations on non-experts,” International Conference on ArtsIT, Interactivity and Game Creation (Springer), 457–467. 10.1007/978-3-030-95531-1_31

[B98] Selten F. Robeer M. Grimmelikhuijsen S. (2023). ‘Just like I thought': street-level bureaucrats trust AI recommendations if they confirm their professional judgment. Public Adm. Rev. 83, 263–278. 10.1111/puar.13602

[B99] Souza J. Leung C. K. (2021). “Explainable artificial intelligence for predictive analytics on customer turnover: a user-friendly interface for non-expert users,” in Explainable AI Within the Digital Transformation and Cyber Physical Systems: XAI Methods and Applications (Cham: Springer), 47–67. 10.1007/978-3-030-76409-8_4

[B100] Swamy V. Du S. Marras M. Kaser T. (2023). “Trusting the explainers: teacher validation of explainable artificial intelligence for course design,” in LAK23: 13th International Learning Analytics and Knowledge Conference (New York, NY: ACM), 345–356. 10.1145/3576050.3576147

[B101] Umbrello S. Yampolskiy R. V. (2022). Designing AI for explainability and verifiability: a value sensitive design approach to avoid artificial stupidity in autonomous vehicles. Int. J. Soc. Robot. 14, 313–322. 10.1007/s12369-021-00790-w

[B102] Upasane S. J. Hagras H. Anisi M. H. Savill S. Taylor I. Manousakis K. . (2024). A type-2 fuzzy based explainable AI system for predictive maintenance within the water pumping industry. IEEE Trans. Artif. Intell. 5, 490–504. 10.1109/TAI.2023.3279808

[B103] van der Waa J. Schoonderwoerd T. van Diggelen J. Neerincx M. (2020). Interpretable confidence measures for decision support systems. Int. J. Hum. Comput. Stud. 144:102493. 10.1016/j.ijhcs.2020.102493

[B104] Veldhuis M. S. Ariëns S. Ypma R. J. Abeel T. Benschop C. C. (2022). Explainable artificial intelligence in forensics: realistic explanations for number of contributor predictions of DNA profiles. Forensic Sci. Int. Genet. 56:102632. 10.1016/j.fsigen.2021.102632 34839075

[B105] Venkatesh V. Bala H. (2008). Technology acceptance model 3 and a research agenda on interventions. Decis. Sci. 39, 273–315. 10.1111/j.1540-5915.2008.00192.x

[B106] Venkatesh V. Morris M. G. Davis G. B. Davis F. D. (2003). User acceptance of information technology: toward a unified view. MIS Q. 27, 425–478. 10.2307/3003654038408043

[B107] Vilone G. Longo L. (2021). Notions of explainability and evaluation approaches for explainable artificial intelligence. Inf. Fusion 76, 89–106. 10.1016/j.inffus.2021.05.00934844219

[B108] Wang Q. Huang K. Chandak P. Zitnik M. Gehlenborg N. (2022a). Extending the nested model for user-centric XAI: a design study on GNN-based drug repurposing. IEEE Trans. Vis. Comput. Graph. 29, 1266–1276. 10.1109/TVCG.2022.3209435 36223348

[B109] Wang X. Yin M. (2021). “Are explanations helpful? A comparative study of the effects of explanations in AI-assisted decision-making,” in 26th international conference on intelligent user interfaces (New York, NY: ACM), 318–328. 10.1145/3397481.3450650

[B110] Wang Y. Venkatesh P. Lim B. Y. (2022b). “Interpretable directed diversity: leveraging model explanations for iterative crowd ideation,” in Proceedings of the 2022 CHI Conference on Human Factors in Computing Systems (New York, NY: ACM), 1–28. 10.1145/3491102.3517551

[B111] Warren G. Keane M. T. Byrne R. M. (2022). Features of explainability: how users understand counterfactual and causal explanations for categorical and continuous features in XAI. arXiv [Preprint]. arXiv:2204.10152. 10.48550/arXiv.2204.10152

[B112] Weitz K. Schiller D. Schlagowski R. Huber T. André E. (2021). “Let me explain!”: exploring the potential of virtual agents in explainable AI interaction design. J. Multimodal User Interfaces 15, 87–98. 10.1007/s12193-020-00332-0

[B113] Wysocki O. Davies J. K. Vigo M. Armstrong A. C. Landers D. Lee R. . (2023). Assessing the communication gap between AI models and healthcare professionals: explainability, utility and trust in AI-driven clinical decision-making. Artif. Intell. 316:103839. 10.1016/j.artint.2022.103839 PMC7618637 41550460

[B114] Xu Y. Collenette J. Dennis L. Dixon C. (2023). “Dialogue explanations for rule-based AI systems,” in International Workshop on Explainable, Transparent Autonomous Agents and Multi-Agent Systems (Cham: Springer), 59–77. 10.1007/978-3-031-40878-6_4

[B115] Zhou J. Gandomi A. H. Chen F. Holzinger A. (2021). Evaluating the quality of machine learning explanations: a survey on methods and metrics. Electronics 10:593. 10.3390/electronics1005059338825185

[B116] Žlahtič B. Završnik J. Blažun Vošner H. Kokol P. Šuran D. Završnik T. (2023). Agile machine learning model development using data canyons in medicine: a step towards explainable artificial intelligence and flexible expert-based model improvement. Appl. Sci. 13:8329. 10.3390/app13148329

[B117] Zöller M.-A. Titov W. Schlegel T. Huber M. F. (2023). XAutoML: a visual analytics tool for understanding and validating automated machine learning. ACM Trans. Interact. Intell. Syst. 13, 1–39. 10.1145/3625240

