# Supplementary material for: Human-centered evaluation of explainable AI applications: a systematic review
Source: Front Artif Intell. 2024 Oct 17;7:1456486. doi: 10.3389/frai.2024.1456486 (PMC11525002; doi:10.3389/frai.2024.1456486)
Supplement: Supplementary file 1 [file Data_Sheet_1.PDF]

Supplementary Material

1 TAXONOMY OF HUMAN-CENTERED XAI EVALUATION: PROPERTIES AND METRICS

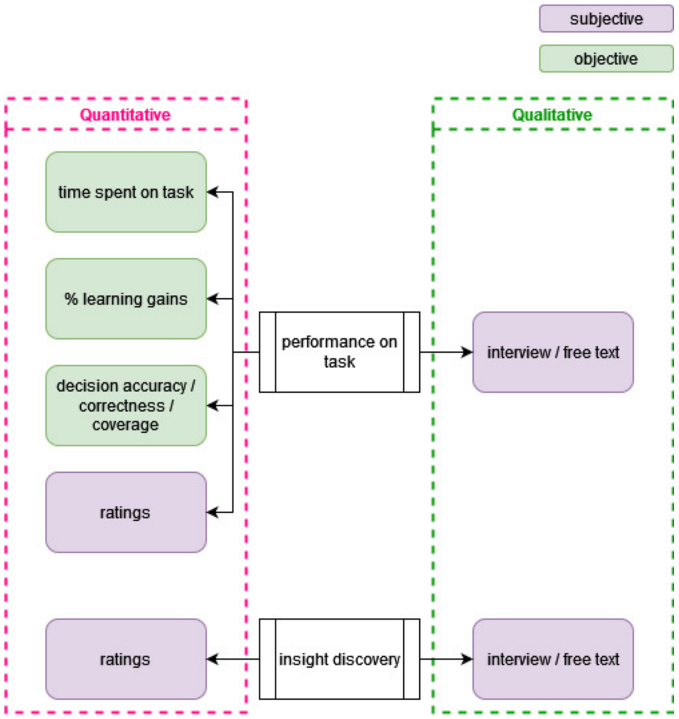

Figure S1. Taxonomy of evaluation properties and metrics related to human-AI performance.

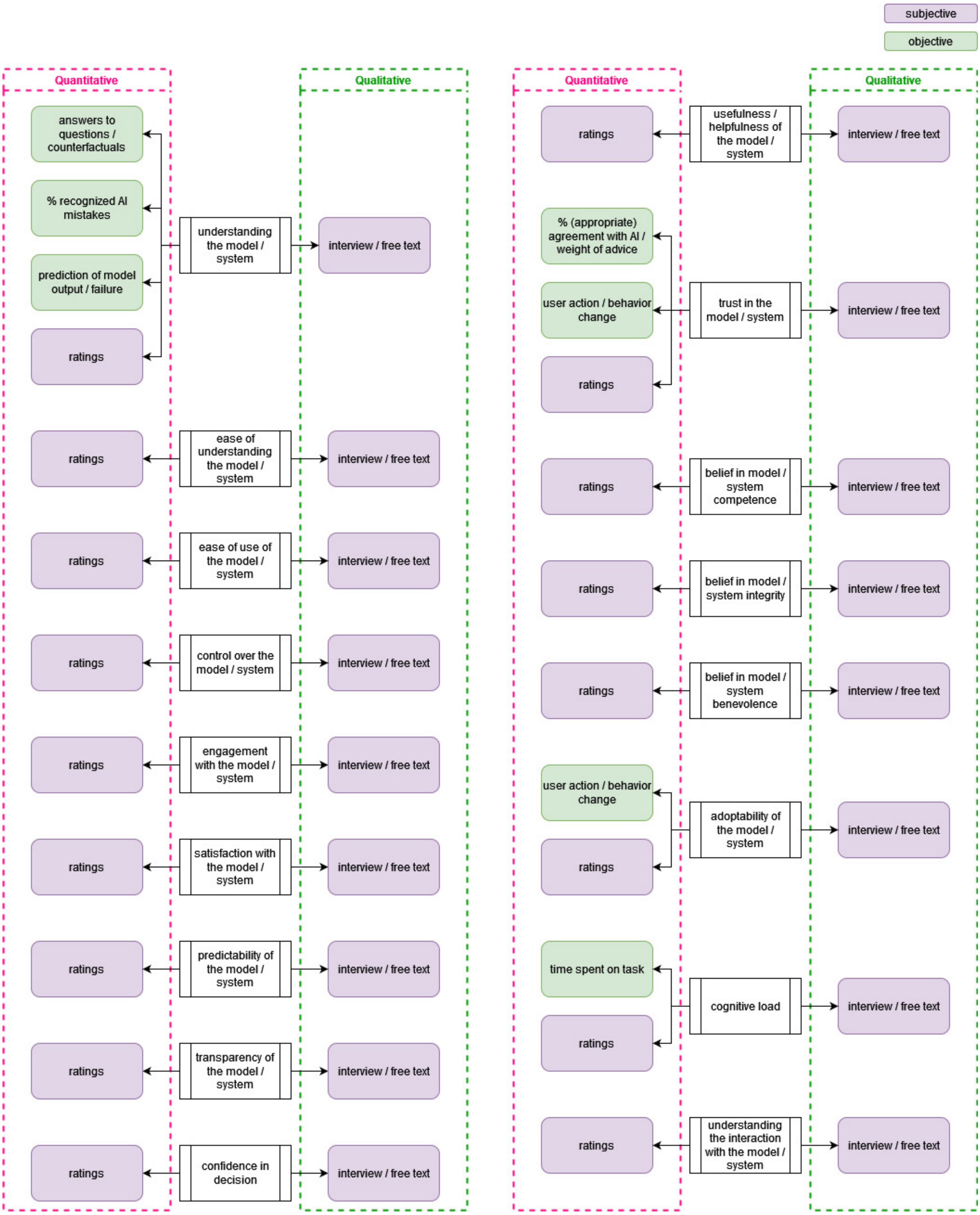

Figure S2. Taxonomy of evaluation properties and metrics related to the quality of human-AI interaction.

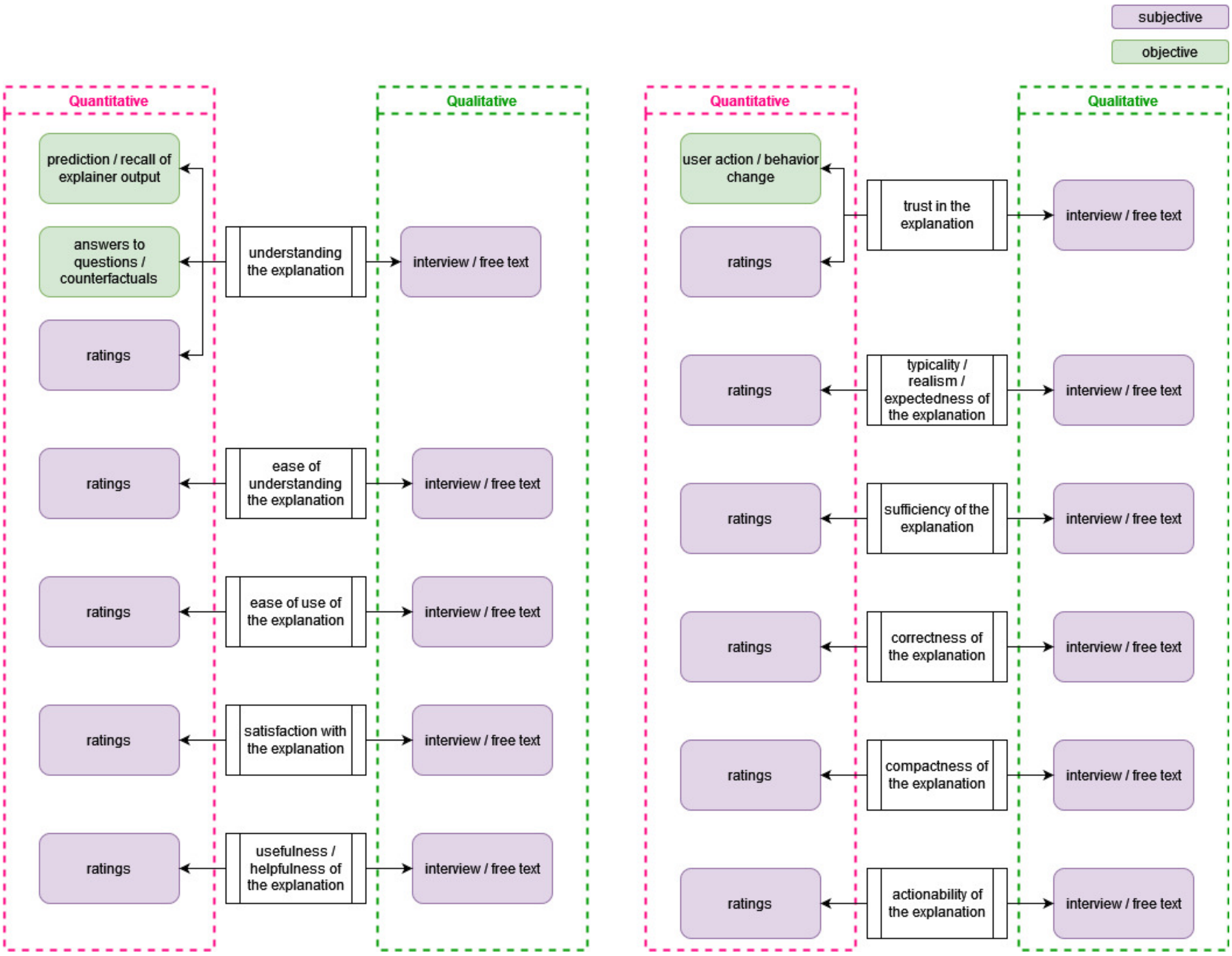

Figure S3. Taxonomy of evaluation properties and metrics related to in-context quality of explanations.

2 DISTRIBUTION OF EVALUATION METRICS IN THE PAPERS

Table S1. Distribution of human-AI performance evaluation metrics in the papers

property	metric	papers
insight discovery	interview / free text	Eriksson and Grov (2022); Maltbie et al. (2021)
performance on task	decision accuracy / correctness / coverage	Bansal et al. (2021); Buçinca et al. (2020); Confalonieri et al. (2021); Ghai et al. (2021); Ibrahim et al. (2023); La Gatta et al. (2021a); La Gatta et al. (2021b); Nazaretsky et al. (2022); Wang et al. (2022a); Alufaisan et al. (2021); Cau et al. (2023); Naiseh et al. (2023); Wang et al. (2022b)
	percentage learning gains	Conati et al. (2021)
	ratings	Jmoona et al. (2023)
	time spent on task	Raab et al. (2023); Confalonieri et al. (2021); Wang et al. (2022a); Schrills and Franke (2023); Wang et al. (2022b)

Table S2. Distribution of explanation quality evaluation metrics in the papers

property	metric	papers
actionability of the explanation	interview / free text	Bhattacharya et al. (2023);
compactness of the explanation	ratings	Moradi and Samwald (2021)
correctness of the explanation	interview / free text	Maltbie et al. (2021)
	ratings	Meas et al. (2022)
ease of understanding the explanation	ratings	Xu et al. (2023); Wang et al. (2022b); Abdul et al. (2020)
ease of use of the explanation	ratings	Jang et al. (2023); Veldhuis et al. (2022); Wang et al. (2022b)
satisfaction with explanation	interview / free text	van der Waa et al. (2020)
	ratings	Brdnik et al. (2023); Hernandez-Bocanegra and Ziegler (2023); Kim et al. (2023); Schellingerhout et al. (2022); Schulze-Weddige and Zylowski (2021); van der Waa et al. (2020); Avetisyan et al. (2022); Guo et al. (2022); Meas et al. (2022); Warren et al. (2022); Ben David et al. (2021); Conijn et al. (2023); Khodabandehloo et al. (2021); Das et al. (2023); Veldhuis et al. (2022); Aechtner et al. (2022); Buçinca et al. (2020); Panigutti et al. (2022); Panigutti et al. (2023); Schrills and Franke (2023)
sufficiency of the explanation	interview / free text	Weitz et al. (2021)
	ratings	Adhikari et al. (2019); Das et al. (2023); Hernandez-Bocanegra and Ziegler (2023); Weitz et al. (2021); Zöller et al. (2023); Aechtner et al. (2022); Fernandes et al. (2023); Ooge et al. (2022); Veldhuis et al. (2022); Chien et al. (2022)
trust in the explanation	interview / free text	Swamy et al. (2023)
	ratings	Polley et al. (2021); Bhattacharya et al. (2023); Brdnik et al. (2023); Fernandes et al. (2023); Warren et al. (2022)
	user action / behavior change	Das et al. (2023)
typicality / realism / expectedness of the explanation	ratings	Förster et al. (2021); Jmoona et al. (2023); Neves et al. (2021); Adhikari et al. (2019)
understanding of the explanation	interview / free text	Anjara et al. (2023); Eriksson and Grov (2022); Larasati (2022)
	ratings	Bhattacharya et al. (2023); Moradi and Samwald (2021); Nagy and Molontay (2023); Žlahtič et al. (2023); Jmoona et al. (2023); Confalonieri et al. (2021); Raab et al. (2023)
	user answers to questions / counterfactuals	Bertrand et al. (2023); van der Waa et al. (2020)
	user predictions / recall of explainer output	Abdul et al. (2020); Zöller et al. (2023)
usefulness / helpfulness of the explanation	interview / free text	Scheers and De Laet (2021); Deo and Sontakke (2021)
	ratings	Neves et al. (2021); Wang et al. (2022b); Aechtner et al. (2022); Bhattacharya et al. (2023); Ibrahim et al. (2023); Xu et al. (2023); Bansal et al. (2021); Bhattacharya et al. (2023); Jang et al. (2023); Moradi and Samwald (2021)

Table S3. Distribution of human-AI interaction evaluation metrics in the papers (part 1)

property	metric	papers
adoptability of the model/system	interview / free text	Bhattacharya et al. (2023)
	ratings	Conati et al. (2021); Panigutti et al. (2022); Panigutti et al. (2023); Bayer et al. (2022); Bunde (2021); Ooge et al. (2022)
	user action / behavior change	Ben David et al. (2021); Bayer et al. (2022)
belief in model/system benevolence	ratings	Ooge et al. (2022); Bayer et al. (2022); Chien et al. (2022)
belief in model/system competence	interview / free text	Anjara et al. (2023)
	ratings	Bayer et al. (2022); Chien et al. (2022); Das et al. (2023); Kühnlenz and Kühnlenz (2023); Ooge et al. (2022); Buçinca et al. (2020); Kühnlenz and Kühnlenz (2023); Naiseh et al. (2023)
belief in model/system integrity	ratings	Bayer et al. (2022); Chien et al. (2022); Ooge et al. (2022)
cognitive load	interview / free text	Anjara et al. (2023)
	ratings	Abdul et al. (2020); Bertrand et al. (2023); Avetisyan et al. (2022); Buçinca et al. (2020); Ghai et al. (2021); Schrills and Franke (2023); Chien et al. (2022)
	time spent on task	Abdul et al. (2020); Conijn et al. (2023)
confidence in decision	ratings	Alufaisan et al. (2021); Panigutti et al. (2022); Panigutti et al. (2023); Raab et al. (2023); Veldhuis et al. (2022); Adhikari et al. (2019); Confalonieri et al. (2021); Ibrahim et al. (2023); Wang et al. (2022a); Fernandes et al. (2023)
control over the model/system	ratings	Guo et al. (2022)
ease of understanding the model/system	ratings	Chien et al. (2022)
ease of use of the model/system	ratings	Bunde (2021)
engagement with the model/system	ratings	Bertrand et al. (2023)
predictability of the model/system	ratings	Schrills and Franke (2023); Fernandes et al. (2023)
satisfaction with the model/system	ratings	Wysocki et al. (2023); Conati et al. (2021); Ghai et al. (2021); Guo et al. (2022)
transparency of the model/system	ratings	Schrills and Franke (2023)

Table S4. Distribution of human-AI interaction evaluation metrics in the papers (part 2)

property	metric	papers
trust in the model/system	interview / free text	Deo and Sontakke (2021)
	percentage (appropriate) agreement with AI / weight of advice	Fernandes et al. (2023); Panigutti et al. (2022); Panigutti et al. (2023); Conijn et al. (2023); Cau et al. (2023); Wang and Yin (2021); Naiseh et al. (2023); Bertrand et al. (2023)
	ratings	Avetisyan et al. (2022); Conijn et al. (2023); Kartikeya (2022); Khodabandehloo et al. (2021); Lundberg et al. (2022); Reeder et al. (2023); Selten et al. (2023); Upasane et al. (2023); Veldhuis et al. (2022); Weitz et al. (2021); Ben David et al. (2021); Branley-Bell et al. (2020); Faulhaber et al. (2021); Fernandes et al. (2023); Fu and Tantithamthavorn (2022); Ghai et al. (2021); Naiseh et al. (2023); Wysocki et al. (2023); Aechtner et al. (2022); Buçinca et al. (2020); Bunde (2021); Kühnlenz and Kühnlenz (2023); Raab et al. (2023); Zöller et al. (2023); Bertrand et al. (2023); Kühnlenz and Kühnlenz (2023); Panigutti et al. (2022); Panigutti et al. (2023); Hernandez-Bocanegra and Ziegler (2023); Conati et al. (2021); Ooge et al. (2022); Schrills and Franke (2023); Chien et al. (2022)
	user action / behavior change	Faulhaber et al. (2021); Kartikeya (2022); Selten et al. (2023)
understanding of the model/system	interview / free text	Anjara et al. (2023)
	percentage recognized AI mistakes	Naiseh et al. (2023)
	ratings	Adhikari et al. (2019); Aechtner et al. (2022); Branley-Bell et al. (2020); Naiseh et al. (2023); Zöller et al. (2023); Buçinca et al. (2020); Larasati (2022); Lundberg et al. (2022); Moradi and Samwald (2021); Reeder et al. (2023); Schrills and Franke (2023); Wang and Yin (2021); Hernandez-Bocanegra and Ziegler (2023); Jmoona et al. (2023); Wysocki et al. (2023); Deo and Sontakke (2021); Chien et al. (2022); Conati et al. (2021)
	user answers to questions / counterfactuals	Deo and Sontakke (2021); Wang and Yin (2021); Bertrand et al. (2023); Abdul et al. (2020)
	user predictions of model output / failure	Buçinca et al. (2020); Jang et al. (2023); Larasati (2022); Okumura and Nagao (2023); Warren et al. (2022); Adhikari et al. (2019); Schrills and Franke (2023)
understanding the interaction with the model/system	ratings	Hernandez-Bocanegra and Ziegler (2023)
usefulness / helpfulness of the model/system	interview / free text	Neves et al. (2021); Anjara et al. (2023)
	ratings	Bunde (2021); Cau et al. (2023); Fu and Tantithamthavorn (2022); Bansal et al. (2021); Khodabandehloo et al. (2021); Schellingerhout et al. (2022); Buçinca et al. (2020); Conati et al. (2021); Hernandez-Bocanegra and Ziegler (2023)

3 DISTRIBUTION OF EXPLANATION TYPES IN THE PAPERS

Table S5. Explanation types found in the papers

Explanation Type	Description	Real / Mock-up	# papers
feature importance	Scores that indicate feature relevance, feature contribution or attribution. For example, LIME, SHAP, Layer-Wise Relevance Propagation (LRP), coefficients of logistic regression.	real	44
rule-based	Rule-based systems, decision trees, anchors, graphs.	real	20
		mock-up	10
counterfactuals	“What if?” explanations that show how changing the value of input feature(s) changes the outcome.	real	10
		mock-up	2
model specifications	Performance metrics (e.g. accuracy, F1-score), confusion matrix, confidence score, hyperparameter values.	real	9
		mock-up	2
data specifications	Information about the overall training data (e.g. distribution of feature values, gold labels), or the input values for a specific instant.	real	6
similar examples	Nearest neighbors, similar examples with the same prediction.	real	6
		mock-up	1
dependence plots	Plot showing relations or interactions between features or between feature(s) and outcome. For example, partial dependence plots (PDP), generalized additive model (GAM).	real	4
contrastive examples	Similar examples with a different prediction.	real	2

Table S6. Distribution of explanation types in the papers

Explanation Type	Papers
feature importance	Adhikari et al. (2019); Aechtner et al. (2022); Alufaisan et al. (2021); Bansal et al. (2021); Bertrand et al. (2023); Bhattacharya et al. (2023); Brdnik et al. (2023); Bunde (2021); Cau et al. (2023); Chien et al. (2022); Das et al. (2023); Deo and Sontakke (2021); Eriksson and Grov (2022); Fernandes et al. (2023); Fu and Tantithamthavorn (2022); Ghai et al. (2021); Ibrahim et al. (2023); Jang et al. (2023); Jmoona et al. (2023); Kartikeya (2022); La Gatta et al. (2021b); Larasati (2022); Lundberg et al. (2022); Maltbie et al. (2021); Meas et al. (2022); Moradi and Samwald (2021); Nagy and Molontay (2023); Naiseh et al. (2023); Neves et al. (2021); Panigutti et al. (2022); Panigutti et al. (2023); Polley et al. (2021); Raab et al. (2023); Scheers and De Laet (2021); Schellingerhout et al. (2022); Schulze-Weddige and Zylowski (2021); Swamy et al. (2023); Veldhuis et al. (2022); Wang and Yin (2021); Wang et al. (2022b); Weitz et al. (2021); Wysocki et al. (2023); Žlahtič et al. (2023); Zöller et al. (2023)
rule-based	Bayer et al. (2022); Branley-Bell et al. (2020); Cau et al. (2023); Conati et al. (2021); Confalonieri et al. (2021); Das et al. (2023); Deo and Sontakke (2021); Fernandes et al. (2023); Guo et al. (2022); Khodabandehloo et al. (2021); La Gatta et al. (2021a); Larasati (2022); Maltbie et al. (2021); Nazaretsky et al. (2022); Ooge et al. (2022); Reeder et al. (2023); Upasane et al. (2023); van der Waa et al. (2020); Wang et al. (2022a); Xu et al. (2023)
rule-based (mock-up)	Avetisyan et al. (2022); Ben David et al. (2021); Buçinca et al. (2020); Conijn et al. (2023); Faulhaber et al. (2021); Hernandez-Bocanegra and Ziegler (2023); Kim et al. (2023); Kühnlenz and Kühnlenz (2023); Selten et al. (2023); Warren et al. (2022)
counterfactuals	Bhattacharya et al. (2023); Cau et al. (2023); Förster et al. (2021); Ibrahim et al. (2023); La Gatta et al. (2021b); Naiseh et al. (2023); Okumura and Nagao (2023); Veldhuis et al. (2022); Wang and Yin (2021); Wang et al. (2022b)
counterfactuals (mock-up)	Kim et al. (2023); Warren et al. (2022)
model specifications	Bansal et al. (2021); Branley-Bell et al. (2020); Brdnik et al. (2023); Bunde (2021); Chien et al. (2022); Deo and Sontakke (2021); Kartikeya (2022); Wysocki et al. (2023); Zöller et al. (2023)
model specifications (mock-up)	Ben David et al. (2021); Conijn et al. (2023)
data specifications	Bhattacharya et al. (2023); Branley-Bell et al. (2020); Brdnik et al. (2023); Ooge et al. (2022); Schrills and Franke (2023); Wysocki et al. (2023)
similar examples	Adhikari et al. (2019); Anjara et al. (2023); Cau et al. (2023); Fu and Tantithamthavorn (2022); Naiseh et al. (2023); Wang and Yin (2021)
similar examples (mock-up)	Buçinca et al. (2020)
dependence plots	Abdul et al. (2020); Aechtner et al. (2022); Nagy and Molontay (2023); Zöller et al. (2023)
contrastive examples	Adhikari et al. (2019); Wang et al. (2022b)

REFERENCES

Abdul, A., von der Weth, C., Kankanhalli, M., and Lim, B. Y. (2020). COGAM: measuring and moderating cognitive load in machine learning model explanations. In *Proceedings of the 2020 CHI Conference on Human Factors in Computing Systems*. 1–14

Adhikari, A., Tax, D. M., Satta, R., and Faeth, M. (2019). LEAFAGE: Example-based and feature importance-based explanations for black-box ML models. In *2019 IEEE international conference on fuzzy systems (FUZZ-IEEE)* (IEEE), 1–7

Aechtner, J., Cabrera, L., Katwal, D., Onghena, P., Valenzuela, D. P., and Wilbik, A. (2022). Comparing user perception of explanations developed with XAI methods. In *2022 IEEE International Conference on Fuzzy Systems (FUZZ-IEEE)* (IEEE), 1–7

Alufaisan, Y., Marusich, L. R., Bakdash, J. Z., Zhou, Y., and Kantarcioglu, M. (2021). Does explainable artificial intelligence improve human decision-making? In *Proceedings of the AAAI Conference on Artificial Intelligence*. vol. 35, 6618–6626

Anjara, S. G., Janik, A., Dunford-Stenger, A., Mc Kenzie, K., Collazo-Lorduy, A., Torrente, M., et al. (2023). Examining explainable clinical decision support systems with think aloud protocols. *Plos one* 18, e0291443

Avetisyan, L., Ayoub, J., and Zhou, F. (2022). Investigating explanations in conditional and highly automated driving: The effects of situation awareness and modality. *Transportation research part F: traffic psychology and behaviour* 89, 456–466

Bansal, G., Wu, T., Zhou, J., Fok, R., Nushi, B., Kamar, E., et al. (2021). Does the whole exceed its parts? the effect of AI explanations on complementary team performance. In *Proceedings of the 2021 CHI Conference on Human Factors in Computing Systems*. 1–16

Bayer, S., Gimpel, H., and Markgraf, M. (2022). The role of domain expertise in trusting and following explainable AI decision support systems. *Journal of Decision Systems* 32, 110–138

Ben David, D., Resheff, Y. S., and Tron, T. (2021). Explainable ai and adoption of financial algorithmic advisors: an experimental study. In *Proceedings of the 2021 AAAI/ACM Conference on AI, Ethics, and Society*. 390–400

Bertrand, A., Eagan, J. R., and Maxwell, W. (2023). Questioning the ability of feature-based explanations to empower non-experts in robo-advised financial decision-making. In *Proceedings of the 2023 ACM Conference on Fairness, Accountability, and Transparency*. 943–958

Bhattacharya, A., Ooge, J., Stiglic, G., and Verbert, K. (2023). Directive explanations for monitoring the risk of diabetes onset: Introducing directive data-centric explanations and combinations to support what-if explorations. In *Proceedings of the 28th International Conference on Intelligent User Interfaces*. 204–219

Branley-Bell, D., Whitworth, R., and Coventry, L. (2020). User trust and understanding of explainable AI: Exploring algorithm visualisations and user biases. In *International Conference on Human-Computer Interaction* (Springer), 382–399

Brdnik, S., Podgorelec, V., and Šumak, B. (2023). Assessing perceived trust and satisfaction with multiple explanation techniques in XAI-enhanced learning analytics. *Electronics* 12, 2594

Buçinca, Z., Lin, P., Gajos, K. Z., and Glassman, E. L. (2020). Proxy tasks and subjective measures can be misleading in evaluating explainable AI systems. In *Proceedings of the 25th international conference on intelligent user interfaces*. 454–464

Bunde, E. (2021). AI-Assisted and explainable hate speech detection for social media moderators–a design science approach

Cau, F. M., Hauptmann, H., Spano, L. D., and Tintarev, N. (2023). Supporting high-uncertainty decisions through AI and logic-style explanations. In *Proceedings of the 28th International Conference on Intelligent User Interfaces*. 251–263

Chien, S.-Y., Yang, C.-J., and Yu, F. (2022). XFlag: Explainable fake news detection model on social media. *International Journal of Human–Computer Interaction* 38, 1808–1827

Conati, C., Barral, O., Putnam, V., and Rieger, L. (2021). Toward personalized XAI: A case study in intelligent tutoring systems. *Artificial intelligence* 298, 103503

Confalonieri, R., Weyde, T., Besold, T. R., and del Prado Martín, F. M. (2021). Using ontologies to enhance human understandability of global post-hoc explanations of black-box models. *Artificial Intelligence* 296, 103471

Conijn, R., Kahr, P., and Snijders, C. (2023). The effects of explanations in automated essay scoring systems on student trust and motivation. *Journal of Learning Analytics* 10, 37–53

Das, D., Nishimura, Y., Vivek, R. P., Takeda, N., Fish, S. T., Ploetz, T., et al. (2023). Explainable activity recognition for smart home systems. *ACM Transactions on Interactive Intelligent Systems* 13, 1–39

Deo, S. and Sontakke, N. (2021). User-centric explainability in fintech applications. In *HCI International 2021-Posters: 23rd HCI International Conference, HCII 2021, Virtual Event, July 24–29, 2021, Proceedings, Part II 23* (Springer), 481–488

Eriksson, H. S. and Grov, G. (2022). Towards xai in the soc–a user centric study of explainable alerts with shap and lime. In *2022 IEEE International Conference on Big Data (Big Data)* (IEEE), 2595–2600

Faulhaber, A. K., Ni, I., and Schmidt, L. (2021). The effect of explanations on trust in an assistance system for public transport users and the role of the propensity to trust. In *Proceedings of Mensch und Computer 2021*. 303–310

Fernandes, G. J., Choi, A., Schauer, J. M., Pfammatter, A. F., Spring, B. J., Darwiche, A., et al. (2023). An Explainable Artificial Intelligence Software Tool for Weight Management Experts (PRIMO): Mixed Methods Study. *Journal of medical Internet research* 25, e42047

Förster, M., Hühn, P., Klier, M., and Kluge, K. (2021). Capturing users' reality: A novel approach to generate coherent counterfactual explanations

Fu, M. and Tantithamthavorn, C. (2022). GPT2SP: A transformer-based agile story point estimation approach. *IEEE Transactions on Software Engineering* 49, 611–625

Ghai, B., Liao, Q. V., Zhang, Y., Bellamy, R., and Mueller, K. (2021). Explainable active learning (XAL) toward AI explanations as interfaces for machine teachers. *Proceedings of the ACM on Human-Computer Interaction* 4, 1–28

Guo, L., Daly, E. M., Alkan, O., Mattetti, M., Cornec, O., and Knijnenburg, B. (2022). Building trust in interactive machine learning via user contributed interpretable rules. In *27th International Conference on Intelligent User Interfaces*. 537–548

Hernandez-Bocanegra, D. C. and Ziegler, J. (2023). Explaining recommendations through conversations: Dialog model and the effects of interface type and degree of interactivity. *ACM Transactions on Interactive Intelligent Systems* 13, 1–47

Ibrahim, L., Ghassemi, M. M., and Alhanai, T. (2023). Do explanations improve the quality of AI-assisted human decisions? an algorithm-in-the-loop analysis of factual & counterfactual explanations. In *Proceedings of the 2023 International Conference on Autonomous Agents and Multiagent Systems*. 326–334

Jang, J., Kim, M., Bui, T.-C., and Li, W.-S. (2023). Toward interpretable machine learning: Constructing polynomial models based on feature interaction trees. In *Pacific-Asia Conference on Knowledge Discovery and Data Mining* (Springer), 159–170

Jmoona, W., Ahmed, M. U., Islam, M. R., Barua, S., Begum, S., Ferreira, A., et al. (2023). Explaining the unexplainable: Role of XAI for flight take-off time delay prediction. In *IFIP International Conference on Artificial Intelligence Applications and Innovations* (Springer), 81–93

Kartikeya, A. (2022). Examining correlation between trust and transparency with explainable artificial intelligence. In *Science and Information Conference* (Springer), 353–358

Khodabandehloo, E., Riboni, D., and Alimohammadi, A. (2021). HealthXAI: Collaborative and explainable AI for supporting early diagnosis of cognitive decline. *Future Generation Computer Systems* 116, 168–189

Kim, D., Song, Y., Kim, S., Lee, S., Wu, Y., Shin, J., et al. (2023). How should the results of artificial intelligence be explained to users?-research on consumer preferences in user-centered explainable artificial intelligence. *Technological Forecasting and Social Change* 188, 122343

Kühnlenz, K. and Kühnlenz, B. (2023). Study on the impact of situational explanations and prior information given to users on trust and perceived intelligence in autonomous driving in a video-based 2x2 design. In *2023 32nd IEEE International Conference on Robot and Human Interactive Communication (RO-MAN)* (IEEE), 1509–1513

La Gatta, V., Moscato, V., Postiglione, M., and Sperli, G. (2021a). CASTLE: Cluster-aided space transformation for local explanations. *Expert Systems with Applications* 179, 115045

La Gatta, V., Moscato, V., Postiglione, M., and Sperlì, G. (2021b). PASTLE: Pivot-aided space transformation for local explanations. *Pattern Recognition Letters* 149, 67–74

Larasati, R. (2022). Explainable AI for breast cancer diagnosis: Application and user's understandability perception. In *2022 International Conference on Electrical, Computer and Energy Technologies (ICECET)* (IEEE), 1–6

Lundberg, H., Mowla, N. I., Abedin, S. F., Thar, K., Mahmood, A., Gidlund, M., et al. (2022). Experimental analysis of trustworthy in-vehicle intrusion detection system using eXplainable Artificial Intelligence (XAI). *IEEE Access* 10, 102831–102841

Maltbie, N., Niu, N., Van Doren, M., and Johnson, R. (2021). XAI tools in the public sector: A case study on predicting combined sewer overflows. In *Proceedings of the 29th ACM Joint Meeting on European Software Engineering Conference and Symposium on the Foundations of Software Engineering*. 1032–1044

Meas, M., Machlev, R., Kose, A., Tepljakov, A., Loo, L., Levron, Y., et al. (2022). Explainability and transparency of classifiers for air-handling unit faults using explainable artificial intelligence (XAI). *Sensors* 22, 6338

Moradi, M. and Samwald, M. (2021). Post-hoc explanation of black-box classifiers using confident itemsets. *Expert Systems with Applications* 165, 113941

Nagy, M. and Molontay, R. (2023). Interpretable dropout prediction: Towards XAI-based personalized intervention. *International Journal of Artificial Intelligence in Education* , 1–27

Naiseh, M., Al-Thani, D., Jiang, N., and Ali, R. (2023). How the different explanation classes impact trust calibration: The case of clinical decision support systems. *International Journal of Human-Computer Studies* 169, 102941

Nazaretsky, T., Bar, C., Walter, M., and Alexandron, G. (2022). Empowering teachers with AI: Co-designing a learning analytics tool for personalized instruction in the science classroom. In *LAK22: 12th International Learning Analytics and Knowledge Conference*. 1–12

Neves, I., Folgado, D., Santos, S., Barandas, M., Campagner, A., Ronzio, L., et al. (2021). Interpretable heartbeat classification using local model-agnostic explanations on ECGs. *Computers in Biology and Medicine* 133, 104393

Okumura, H. and Nagao, T. (2023). MIPCE: Generating multiple patches counterfactual-changing explanations for time series classification. In *International Conference on Artificial Neural Networks* (Springer), 231–242

Ooge, J., Kato, S., and Verbert, K. (2022). Explaining recommendations in e-learning: Effects on adolescents' trust. In *27th International Conference on Intelligent User Interfaces*. 93–105

Panigutti, C., Beretta, A., Fadda, D., Giannotti, F., Pedreschi, D., Perotti, A., et al. (2023). Co-design of human-centered, explainable AI for clinical decision support. *ACM Transactions on Interactive Intelligent Systems* 13, 1–35

Panigutti, C., Beretta, A., Giannotti, F., and Pedreschi, D. (2022). Understanding the impact of explanations on advice-taking: a user study for AI-based clinical decision support systems. In *Proceedings of the 2022 CHI Conference on Human Factors in Computing Systems*. 1–9

Polley, S., Koparde, R. R., Gowri, A. B., Perera, M., and Nuernberger, A. (2021). Towards trustworthiness in the context of explainable search. In *Proceedings of the 44th International ACM SIGIR Conference on Research and Development in Information Retrieval*. 2580–2584

Raab, D., Theissler, A., and Spiliopoulou, M. (2023). XAI4EEG: spectral and spatio-temporal explanation of deep learning-based seizure detection in EEG time series. *Neural Computing and Applications* 35, 10051–10068

Reeder, S., Jensen, J., and Ball, R. (2023). Evaluating explainable AI (XAI) in terms of user gender and educational background. In *International Conference on Human-Computer Interaction* (Springer), 286–304

Scheers, H. and De Laet, T. (2021). Interactive and explainable advising dashboard opens the black box of student success prediction. In *Technology-Enhanced Learning for a Free, Safe, and Sustainable World: 16th European Conference on Technology Enhanced Learning, EC-TEL 2021, Bolzano, Italy, September 20-24, 2021, Proceedings 16* (Springer), 52–66

Schellingerhout, R., Medentsiy, V., and Marx, M. (2022). Explainable career path predictions using neural models

Schrills, T. and Franke, T. (2023). How do users experience traceability of AI systems? examining subjective information processing awareness in automated insulin delivery (AID) systems. *ACM Transactions on Interactive Intelligent Systems* 13, 1–34

Schulze-Weddige, S. and Zylowski, T. (2021). User study on the effects explainable AI visualizations on non-experts. In *International Conference on ArtsIT, Interactivity and Game Creation* (Springer), 457–467

Selten, F., Robeer, M., and Grimmelikhuijsen, S. (2023). 'Just like I thought': Street-level bureaucrats trust AI recommendations if they confirm their professional judgment. *Public Administration Review* 83, 263–278

Swamy, V., Du, S., Marras, M., and Kaser, T. (2023). Trusting the explainers: teacher validation of explainable artificial intelligence for course design. In *LAK23: 13th International Learning Analytics and Knowledge Conference*. 345–356

Upasane, S. J., Hagras, H., Anisi, M. H., Savill, S., Taylor, I., and Manousakis, K. (2023). A type-2 fuzzy based explainable AI system for predictive maintenance within the water pumping industry. *IEEE Transactions on Artificial Intelligence*

van der Waa, J., Schoonderwoerd, T., van Diggelen, J., and Neerincx, M. (2020). Interpretable confidence measures for decision support systems. *International Journal of Human-Computer Studies* 144, 102493

Veldhuis, M. S., Ariëns, S., Ypma, R. J., Abeel, T., and Benschop, C. C. (2022). Explainable artificial intelligence in forensics: Realistic explanations for number of contributor predictions of DNA profiles. *Forensic Science International: Genetics* 56, 102632

Wang, Q., Huang, K., Chandak, P., Zitnik, M., and Gehlenborg, N. (2022a). Extending the nested model for user-centric XAI: A design study on GNN-based drug repurposing. *IEEE Transactions on Visualization and Computer Graphics* 29, 1266–1276

Wang, X. and Yin, M. (2021). Are explanations helpful? a comparative study of the effects of explanations in AI-assisted decision-making. In *26th international conference on intelligent user interfaces*. 318–328

Wang, Y., Venkatesh, P., and Lim, B. Y. (2022b). Interpretable directed diversity: Leveraging model explanations for iterative crowd ideation. In *Proceedings of the 2022 CHI Conference on Human Factors in Computing Systems*. 1–28

Warren, G., Keane, M. T., and Byrne, R. M. (2022). Features of explainability: How users understand counterfactual and causal explanations for categorical and continuous features in XAI. *arXiv preprint arXiv:2204.10152*

Weitz, K., Schiller, D., Schlagowski, R., Huber, T., and André, E. (2021). "Let me explain!": exploring the potential of virtual agents in explainable AI interaction design. *Journal on Multimodal User Interfaces* 15, 87–98

Wysocki, O., Davies, J. K., Vigo, M., Armstrong, A. C., Landers, D., Lee, R., et al. (2023). Assessing the communication gap between AI models and healthcare professionals: Explainability, utility and trust in AI-driven clinical decision-making. *Artificial Intelligence* 316, 103839

Xu, Y., Collenette, J., Dennis, L., and Dixon, C. (2023). Dialogue explanations for rule-based AI systems. In *International Workshop on Explainable, Transparent Autonomous Agents and Multi-Agent Systems* (Springer), 59–77

Žlahtič, B., Završnik, J., Blažun Vošner, H., Kokol, P., Šuran, D., and Završnik, T. (2023). Agile machine learning model development using data canyons in medicine: A step towards explainable artificial intelligence and flexible expert-based model improvement. *Applied Sciences* 13, 8329

Zöller, M.-A., Titov, W., Schlegel, T., and Huber, M. F. (2023). XAutoML: A visual analytics tool for understanding and validating automated machine learning. *ACM Transactions on Interactive Intelligent Systems* 13, 1–39
