# Supplementary figures and images for: Human-centered evaluation of explainable AI applications: a systematic review
Source: Front Artif Intell. 2024 Oct 17;7:1456486. doi: 10.3389/frai.2024.1456486 (PMC11525002; doi:10.3389/frai.2024.1456486)

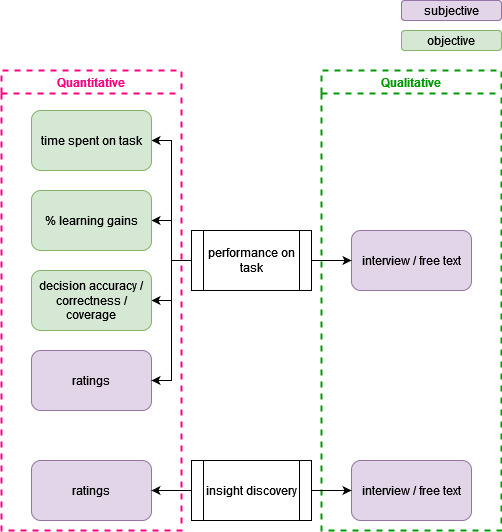

Supplement: Supplementary file 2 [file Data_Sheet_2.ZIP › figures/eval_taxo_performance.jpg]

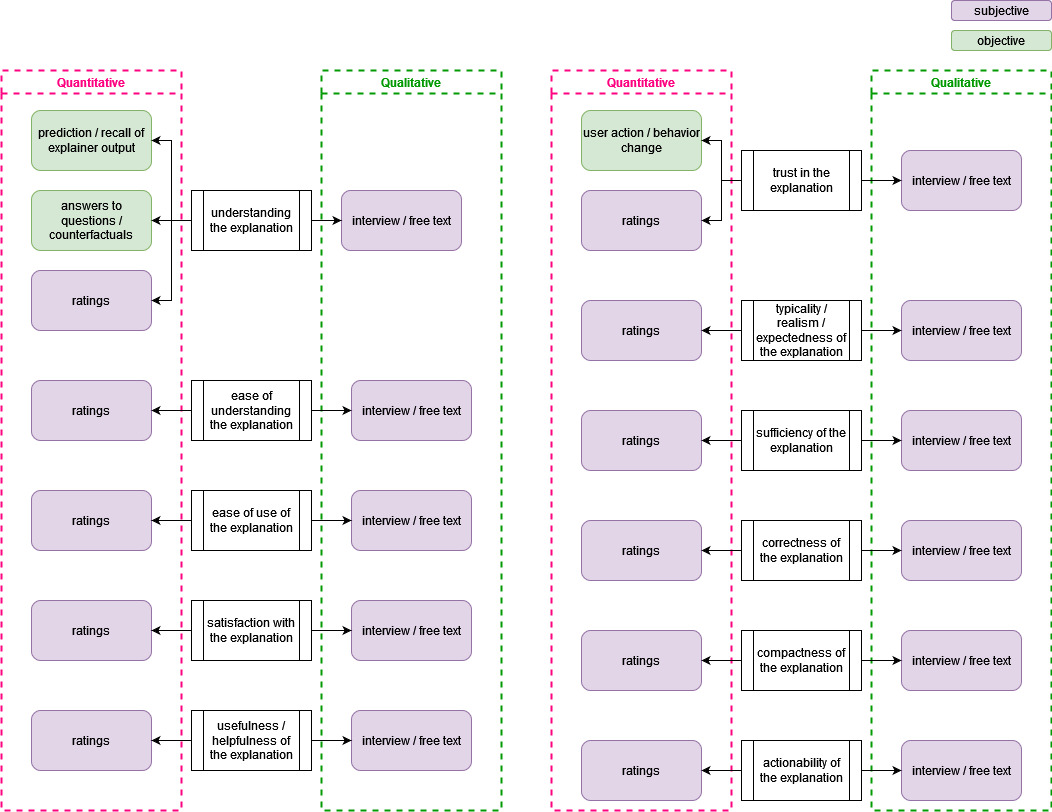

Supplement: Supplementary file 2 [file Data_Sheet_2.ZIP › figures/eval_taxo_explanation.jpg]

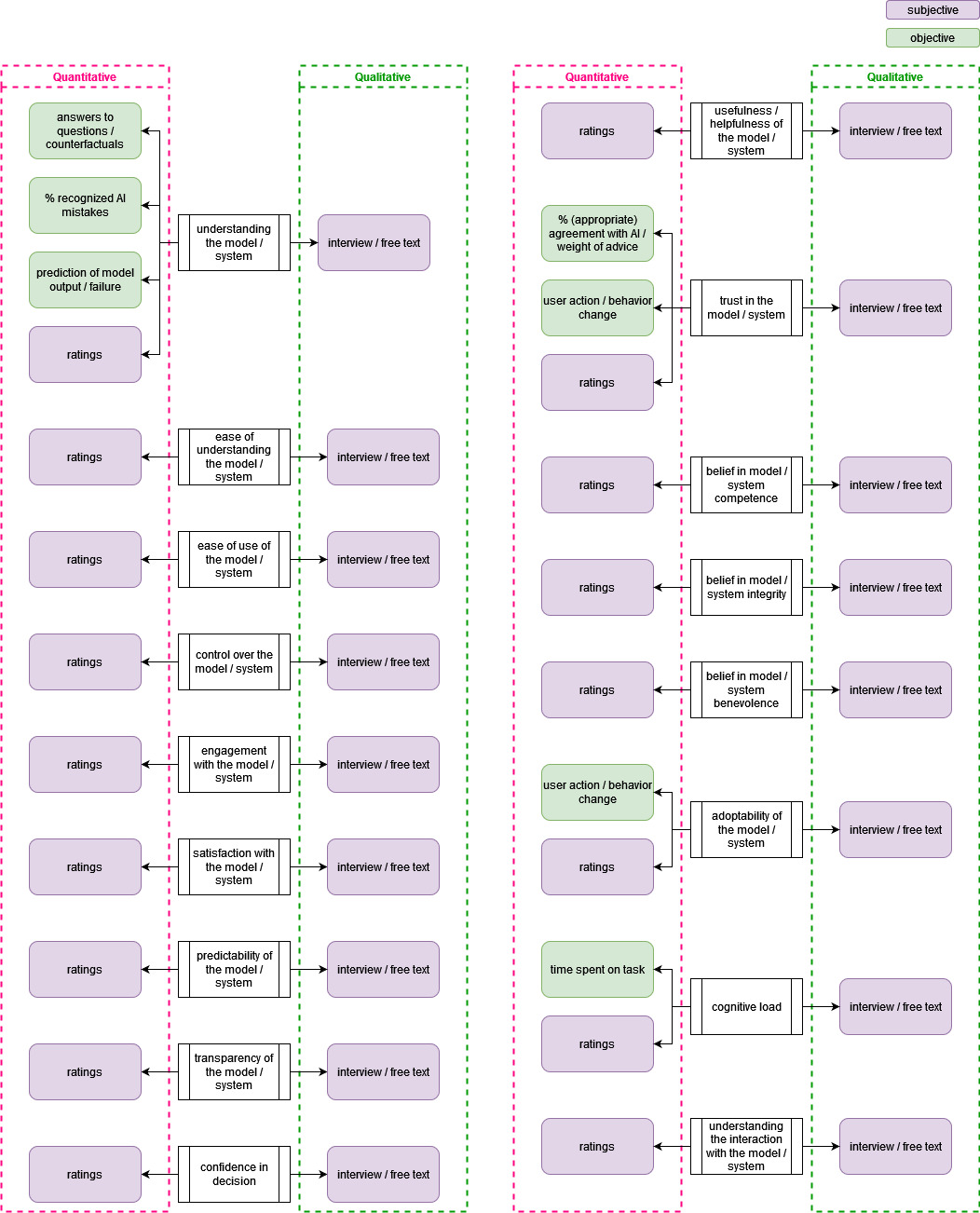

Supplement: Supplementary file 2 [file Data_Sheet_2.ZIP › figures/eval_taxo_interaction.jpg]
